# Neomorphic G**α**o mutations gain interaction with Ric8 proteins in *GNAO1* encephalopathies

**DOI:** 10.1172/JCI172057

**Published:** 2024-06-14

**Authors:** Gonzalo P. Solis, Alexey Koval, Jana Valnohova, Arghavan Kazemzadeh, Mikhail Savitsky, Vladimir L. Katanaev

**Affiliations:** 1 Translational Research Center in Oncohaematology, Department of Cell Physiology and Metabolism, Faculty of Medicine, University of Geneva, Geneva, Switzerland.; 2 School of Medicine and Life Sciences, Department of Pharmacy and Pharmacology, Far Eastern Federal University, Vladivostok, Russia.

**Keywords:** Cell biology, Genetics, G proteins, Genetic diseases, Neurological disorders

## Abstract

*GNAO1* mutated in pediatric encephalopathies encodes the major neuronal G protein Gαo. Of the more than 80 pathogenic mutations, most are single amino acid substitutions spreading across the Gαo sequence. We performed extensive characterization of Gαo mutants, showing abnormal GTP uptake and hydrolysis and deficiencies in binding Gβγ and RGS19. Plasma membrane localization of Gαo was decreased for a subset of mutations that leads to epilepsy; dominant interactions with GPCRs also emerged for the more severe mutants. Pathogenic mutants massively gained interaction with Ric8A and, surprisingly, Ric8B proteins, relocalizing them from cytoplasm to Golgi. Of these 2 mandatory Gα-subunit chaperones, Ric8A is normally responsible for the Gαi/Gαo, Gαq, and Gα12/Gα13 subfamilies, and Ric8B solely responsible for Gαs/Gαolf. Ric8 mediates the disease dominance when engaging in neomorphic interactions with pathogenic Gαo through imbalance of the neuronal G protein signaling networks. As the strength of Gαo-Ric8B interactions correlates with disease severity, our study further identifies an efficient biomarker and predictor for clinical manifestations in *GNAO1* encephalopathies. Our work uncovers the neomorphic molecular mechanism of mutations underlying pediatric encephalopathies and offers insights into other maladies caused by G protein malfunctioning and further genetic diseases.

## Abstract 2

Neomorphic *GNAO1* mutations underlying pediatric encephalopathies inform on other maladies caused by G protein–misfunctioning and other genetic diseases.

## Graphical abstract

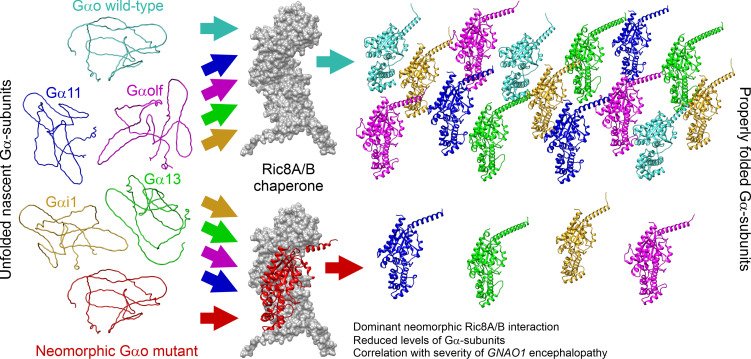

## Introduction

Heterotrimeric G proteins are the principal transducers of G protein–coupled receptors (GPCRs) — the biggest receptor family in animals — and consist of α, β, and γ subunits. Sixteen human Gα subunits fall into Gαi/Gαo, Gαs, Gαq, and Gα12/Gα13 subclasses. Upon activation, the cognate GPCR acts as a guanine-nucleotide exchange factor (GEF), catalyzing the GDP-GTP exchange on Gα and leading to the heterotrimer dissociation into Gα-GTP and Gβγ, both capable of downstream signaling (1). The intrinsic GTPase activity of Gα, further stimulated by regulator of G protein signaling (RGS) proteins (2), leads to GTP hydrolysis and the resultant Gα-GDP can continue to signal by reloading with GTP (3), or reassociates with Gβγ, closing the cycle (1).

Pediatric *GNAO1* encephalopathies are characterized by a spectrum of clinical manifestations, including early-onset epilepsy, motor dysfunctions, developmental delay, intellectual disability, and occasional brain atrophy (4–7). Caused primarily by dominant de novo mutations in *GNAO1*, the gene encoding the major neuronal G protein Gαo, these encephalopathies lack efficient treatments. Missense mutations are most frequently seen in *GNAO1* encephalopathies and spread throughout the Gαo coding sequence, affecting conserved residues with critical functions in the G protein (8). Thus far, pathological Gαo mutations have been described as loss-of-function (9–11), gain-of-function (10, 12, 13), or dominant-negative (9–11, 14, 15) mutations. This versatility of genetic manifestations has led us to propose that *GNAO1* encephalopathy mutations are none of the above, but are instead of a neomorphic nature (10, 16).

The neomorphic concept of *GNAO1* mutations imposes important constraints on the development of therapies (10), and further assumes that a novel mechanism is gained by pathologic Gαo mutants. Here, through a massive characterization of *GNAO1* mutations, we identify a uniform neomorphic feature: a strong gain of interaction, biochemical and cellular, with Ric8A and, more surprisingly, with Ric8B — the mandatory chaperones of all Gα subunits (17). Furthermore, the neomorphic Gαo-Ric8B interaction emerges as a simple biomarker for the disease severity.

## Results

### Clinical assessment of GNAO1 encephalopathy mutants.

As representatives of pathogenic Gαo mutants, the following 16 were studied: G40R, G45E, S47G, D174G, L199P, G203R, R209C, C215Y, A227V, Y231C, Q233P, E237K, E246K, N270H, F275S, and I279N; a recent Q52R mutation (18) was also included in some analyses. The mutants were grouped following the OMIM catalog (https://omim.org/) that classifies *GNAO1* encephalopathy into 2 disorders with distinct clinical manifestations: “developmental and epileptic encephalopathy-17” (DEE17; OMIM #615473) and “neurodevelopmental disorder with involuntary movements” (NEDIM; OMIM #617493) (Supplemental Table 1; supplemental material available online with this article; https://doi.org/10.1172/JCI172057DS1). While motor dysfunction is typically present in both DEE17 and NEDIM, the former additionally includes epilepsy. However, some patients carrying the frequent NEDIM mutations R209C and E246K also suffer from epilepsy (Supplemental Table 1). This, together with the emergence of *GNAO1* mutations associated with a milder dystonia phenotype (19), suggests that *GNAO1*-related disorders might represent a continuous phenotypic spectrum, although the genotype-phenotype correlation is still unclear (20).

Another important category emanating from the clinical data is the disease onset, which we use as the clinical score for individual mutations (Supplemental Table 1). This analysis separates *GNAO1* encephalopathy cases into those with a very early onset (<10 postnatal days; represented by G45E, L199P, F275S, and I279N), early onset (≥10 days, <3 postnatal months; G40R, Q52R, D174G, G203R, A227V, Y231C, and N270H), late onset (≥3 months, <2 postnatal years; S47G, R209C, E237K, and E246K), and very late onset (≥2 postnatal years; C215Y and Q233P) (Supplemental Table 1). All *GNAO1* mutations leading to DEE17 (except for S47G) lay within the very early and early onsets, whereas all NEDIM mutants are within the very late and late onsets. Correlating with the disease severity, the 29 DEE17 patients combined show a median disease onset of approximately 43 days, as opposed to the approximately 569 days for 31 NEDIM patients (Supplemental Figure 1A).

### Biochemical properties of GNAO1 encephalopathy mutants.

**Figure 1:**
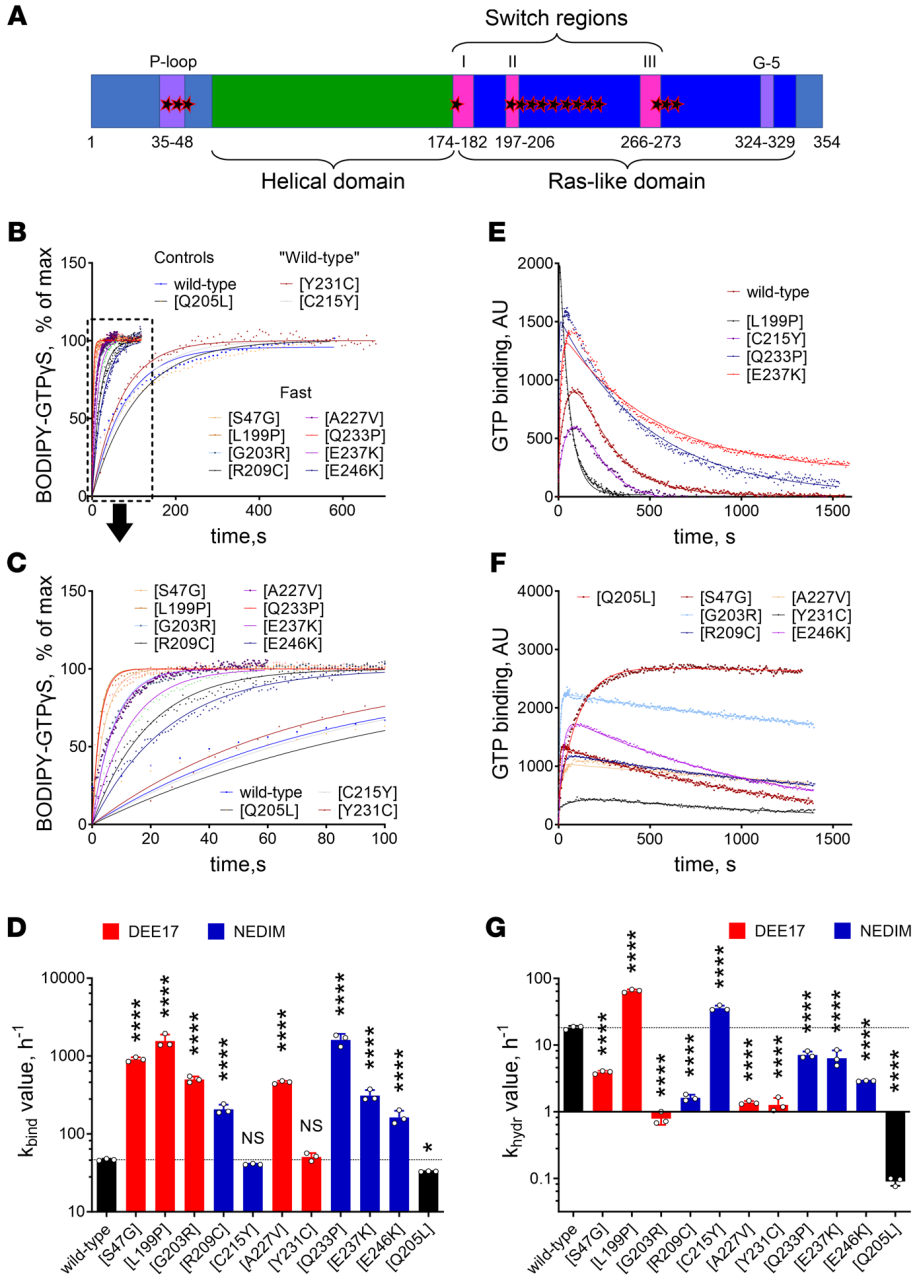
Spectrum of biochemical defects associated with *GNAO1* encephalopathy mutations. (**A**) Scheme of the mutated amino acid residues (stars) in the overall sequence of Gαo. The residues are either located in the P-loop or in the Ras-like domain. (**B** and **C**) Representative curves of BODIPY-GTPγS binding to wild-type Gαo, encephalopathy mutants, and the GTPase-dead Q205L mutant (used as control). Most of the Gαo mutants present strongly elevated binding rates — dotted-line box in **B**, expanded in **C** — whereas only 2 mutants (C215Y and Y231C) display nearly wild-type rates. (**D**) Quantification of the binding rate constant (*k*_bind_) of Gαo variants color-coded according to their association with developmental and epileptic encephalopathy-17 (DEE17; red) or neurodevelopmental disorder with involuntary movements (NEDIM; blue). (**E** and **F**) Representative curves of the course of BODIPY-GTP binding and hydrolysis by wild-type Gαo and active (**E**) or deficient/dead (**F**) mutants. (**G**) Quantification of the hydrolysis rate constant (*k*_hydr_). Note that data are adjusted to the plateau to highlight the differences in the binding rates in **B** and **C**, while raw fluorescence units are shown in **E** and **F**, which are needed for the proper *k*_hydr_ calculation. Data in **D** and **G** represent mean ± SD (*n* = 3). NS, not significant. *P < 0.05, ****P < 0.0001 by 1-way ANOVA followed by Dunnett’s multiple-comparison test.

Gαo mutations affect residues within motifs controlling nucleotide binding and hydrolysis: P-loop, switch regions I, II, and III, and other sites in the Ras-like domain (Figure 1A). Thus, nucleotide uptake/hydrolysis is suspected to be aberrant across *GNAO1* mutations. Indeed, previous studies demonstrated that the Q52P/R mutants displayed complete loss of GTP uptake (18), R209H displayed a faster GTP uptake (21), while G203R, R209C, and E246K displayed faster GTP uptake and lost hydrolysis (10).

We expressed in *Escherichia* *coli* and affinity purified the 16 pathologic Gαo mutants, along with wild-type Gαo and the classical GTPase-dead mutant Q205L as nonpathogenic controls (Supplemental Figure 1B), and studied their GTP uptake/hydrolysis using BODIPY-GTPγS and BODIPY-GTP (3, 10, 22, 23). Six Gαo mutants (G40R, G45E, D174G, N270H, F275S, and I279N) were inactive, as they presented no measurable nucleotide binding capability, similar to the Q52P/R mutants we studied earlier (18). All these mutations, including Q52P/R, lead to the more severe DEE17 disorder (Supplemental Figure 1B and Supplemental Table 1). For the biochemically inactive mutants, the median disease onset of approximately 31 postnatal days (13 patients) is, however, not significantly lower than the approximately 52 days of the remaining 16 DEE17 patients (Supplemental Figure 1C).

Analysis of the biochemically active variants reveals severe abnormalities in GTP uptake and/or hydrolysis. First, the majority revealed a much faster rate of GTP uptake as compared with wild-type Gαo and Q205L (Figure 1, B–D), generalizing the previous findings for G203R, R209C/H, and E246K (10, 21). Of the mutants studied, only C215Y and Y231C demonstrated nearly wild-type rates of GTP uptake; all the others increased the kinetic *k*_bind_ constant from 3.5-fold (E246K) to 34-fold (L199P) (Figure 1D). Second, several Gαo mutants revealed strongly decreased rates of GTP hydrolysis (Figure 1, E–G), again like G203R, R209C, and E246K (10). Only 2 exceptions were seen: the kinetic *k*_hydr_ constant was increased for L199P (3.5-fold) and C215Y (2-fold), while the other mutants showed a drop in *k*_hydr_ from 3-fold (E237K) to 14-fold (Y231C); the GTPase-dead Q205L decreased this constant more than 200-fold (Figure 1G).

Given the strong increase in GTP uptake accompanied by a strong decrease in GTP hydrolysis, mutants are expected to be constitutively GTP loaded. The only exception is C215Y, whose normal GTP uptake and enhanced GTP hydrolysis predict that this variant is preferably GDP loaded compared with wild-type Gαo. To estimate the resulting preponderance of GTP charging, we performed simulations of the GDP-GTP cycling of the Gαo variants using the calculated *k*_bind_ and *k*_hydr_ (see Methods) (24). The ratio of the GTP-loaded to the GDP-bound protein was calculated as 2.56 to 1 for wild-type Gαo. This GTP/GDP ratio was strongly increased among most mutants, from 9.5-fold (L199P) to 245-fold (G203R) — as illustration, a 62.5-fold increase was calculated for Q205L. In contrast, C215Y showed a 2-fold decrease in the GTP/GDP ratio. We found, however, no significant correlation between disease onset and the GTP-loaded proportion of the mutants (Supplemental Figure 1D).

### Cellular characterization of GNAO1 encephalopathy mutants.

Despite these insights into the biochemical properties of Gαo mutants, the complexity of cellular interactions, localizations, and signaling properties exceeds that of purified proteins. Thus, we moved next to massive cellular analyses, using Gαo variants with an internal GFP fusion allowing expression, localization, and coimmunoprecipitation (co-IP) analyses (10, 18). Transfection of the 16 pathologic mutants into the neuroblastoma Neuro-2a (N2a) cell line demonstrated that most Gαo mutants have decreased expression compared with wild-type (Supplemental Figure 2, A and B), with some (G40R, L199P, A227V, Y231C, N270H, and F275S) dropping to as low as approximately 20% of wild-type. Noteworthily, the combined expression for the mutants related to DEE17, approximately 35% of wild-type, was significantly lower than the approximately 50% expression of the NEDIM mutants (Supplemental Figure 2C). In contrast, we found no correlation between disease onset and mutant expression levels (Supplemental Figure 2D).

**Figure 2:**
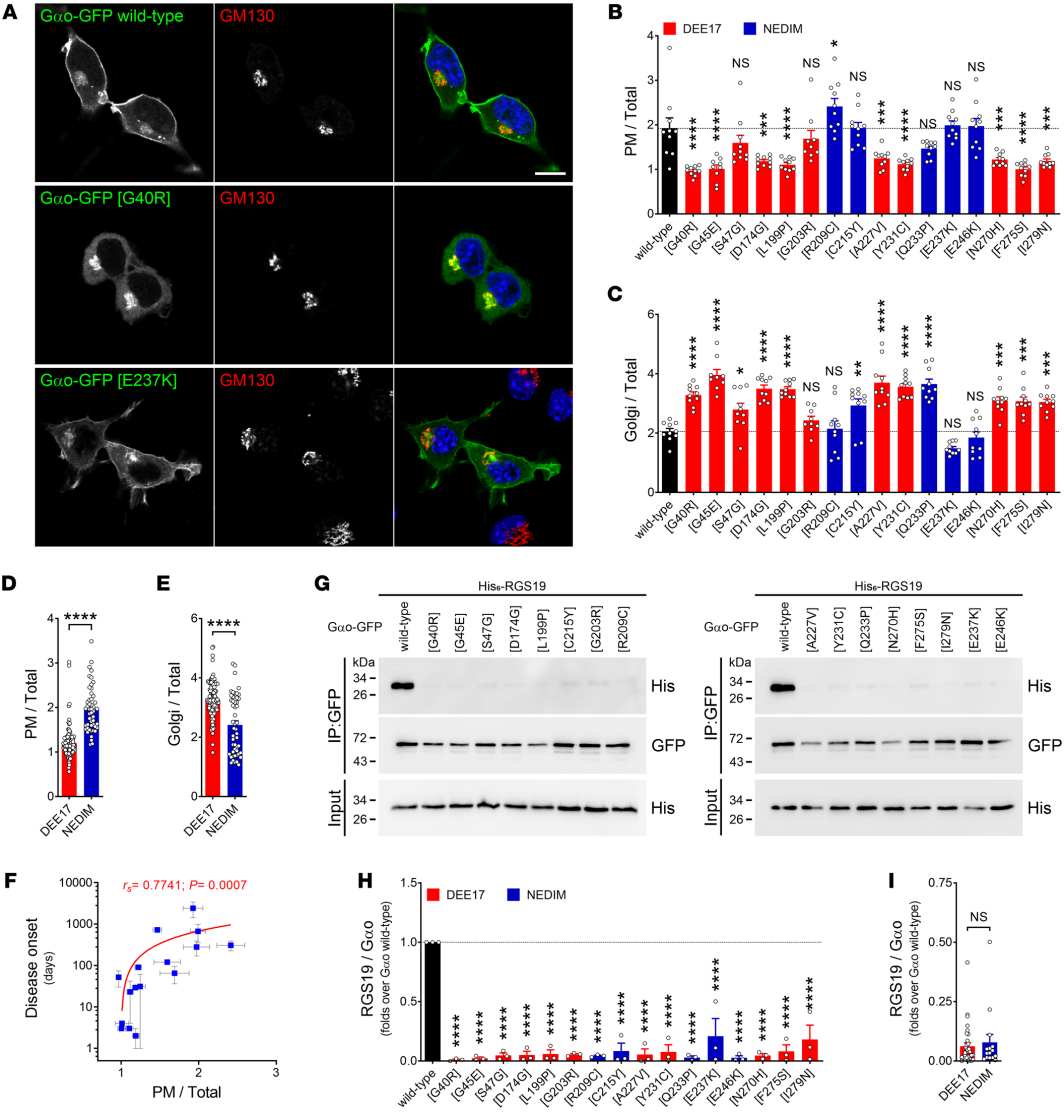
Subcellular localization variations linked to *GNAO1* mutations. (**A**) N2a cells expressing wild-type Gαo-GFP or the encephalopathy mutants G40R and E237K were immunostained against GM130 to visualize the Golgi apparatus and stained with DAPI in blue for nuclei. Scale bar: 10 μm. (**B** and **C**) Mean fluorescence intensity ratios of Gαo-GFP variants at the plasma membrane (PM; **B**) or Golgi (**C**) versus total cell (*n* = 9–10). Bars are color-coded according to the involvement of Gαo mutants in the pathology developmental and epileptic encephalopathy-17 (DEE17; red) or neurodevelopmental disorder with involuntary movements (NEDIM; blue). (**D** and **E**) The combined localization at the PM (**D**) or Golgi (**E**) of the variants connected to DEE17 or NEDIM. (**F**) A scatterplot showing a significant positive correlation between disease onset and PM localization of Gαo mutants. Note the log scale in the *y* axis. (**G**) N2a cells were cotransfected with His_6_-tagged RGS19 and Gαo-GFP variants, and immunoprecipitation (IP) was done with a nanobody against GFP. Co-IP of RGS19 was analyzed by Western blot using antibodies against GFP for Gαo and against the His_6_ tag for RGS19. (**H** and **I**) Quantification of the co-IP of RGS19 by individual Gαo mutants (*n* = 3) (**H**) and pooled in the DEE17 and NEDIM classes (**I**). Data represent mean ± SEM. Data in **B**, **C**, and **H** were analyzed by 1-way ANOVA followed by Dunnett’s multiple-comparison test, in **D**, **E**, and **I** by 2-tailed Mann-Whitney test, and in **F** by 2-tailed Spearman’s correlation test; rank correlation coefficients (*r_s_*) and *P* value are indicated. NS, not significant. *P < 0.05; **P < 0.01; ***P < 0.001; ****P < 0.0001.

Previously, plasma membrane (PM) and Golgi localization of the G203R, R209C, and E246K mutants was observed (10), recapitulating the wild-type Gαo localization (23). In contrast, the Q52P/R mutants revealed severely decreased PM expression with maintained Golgi signal (18). Of note, Golgi localization — with or without PM binding — is indicative of a normal lipidation of Gαo (25). We systematically analyzed the localization pattern of the 16 Gαo mutants, revealing that they fall into 2 major groups. Both groups maintained the Golgi localization, group 1 additionally maintained PM association, while group 2 strongly decreased it (Figure 2, A–C, and Supplemental Figure 3A). A decrease in PM localization correlated significantly with a proportional increase in Golgi localization (Supplemental Figure 3B). Combined, the PM and Golgi localizations of the mutants leading to DEE17 showed significant differences from their NEDIM counterparts (Figure 2, D and E). We also found a significant correlation between disease onset and Gαo mutant localization at the PM, but not at Golgi (Figure 2F and Supplemental Figure 3C). Overall, these findings agree with our initial hypothesis that individual *GNAO1* mutations display DEE17 versus NEDIM phenotypes, depending on which of the 2 Gαo physiologic compartments — PM versus Golgi — is primarily affected by the mutation (26), although some representatives of the DEE17 group, such as S47G and G203R, escape this generalization and do not reveal a significant decrease in PM localization (Figure 2B).

### Interaction of GNAO1 encephalopathy mutants with RGS19 and Gβγ.

We previously showed that the interaction of some Gαo mutants (Q52P/R, G203R, R209C, and E246K) with RGS19, a major regulator of GTP hydrolysis on Gαo (3), is dramatically impaired (10, 18). We have now systematically analyzed RGS19-Gαo binding across the pathogenic mutants through co-IP using an anti-GFP nanobody (10). We see the loss of this interaction as an omnipresent phenomenon for *GNAO1* mutations, equally affecting mutants leading to DEE17 or NEDIM, and regardless of disease onset (Figure 2, G–I, and Supplemental Figure 4A).

**Figure 3:**
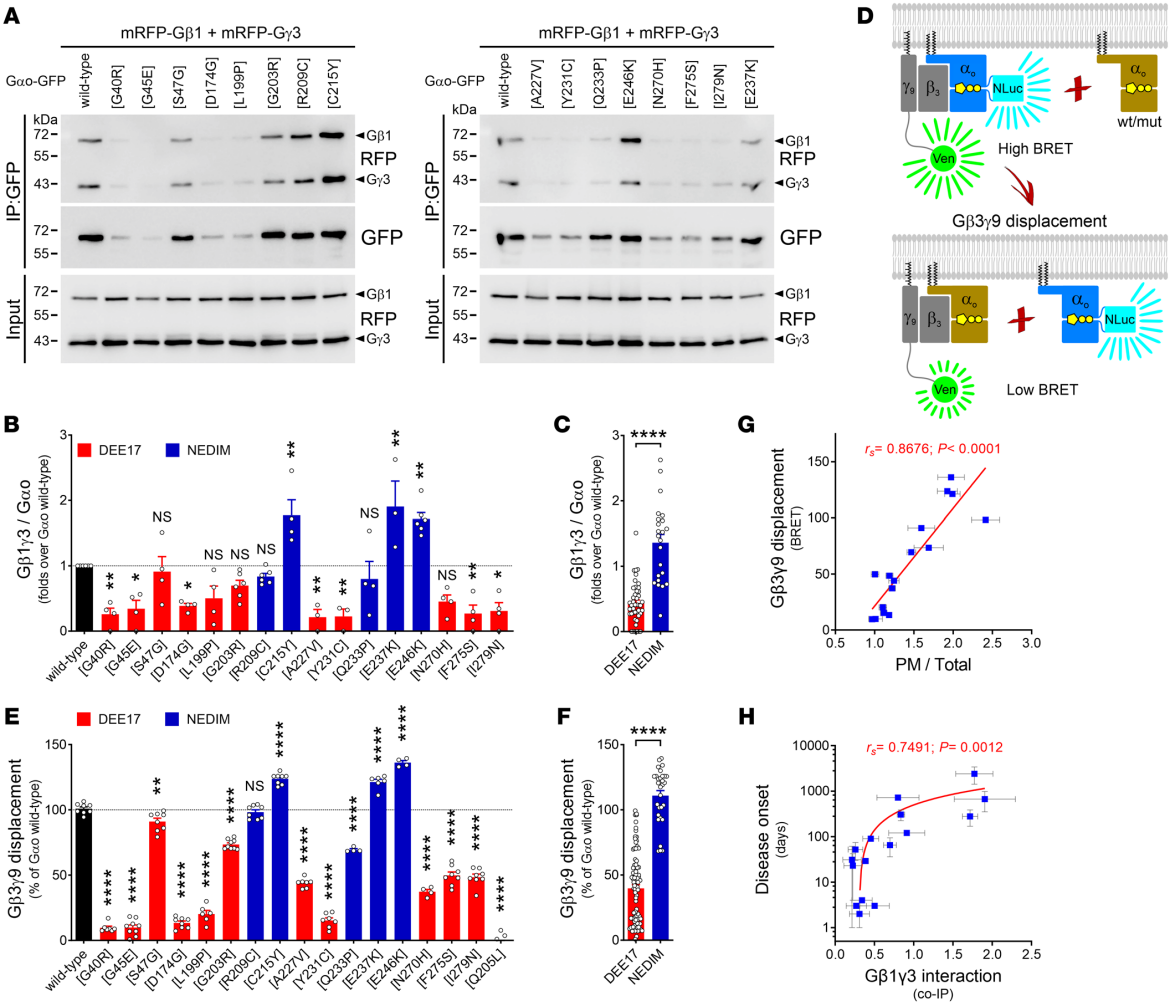
Differential Gβγ binding induced by *GNAO1* mutations. (**A**–**C**) The interaction of Gαo-GFP variants with mRFP-Gβ1 and mRFP-Gγ3 was analyzed by immunoprecipitation (IP) from N2a cells using a nanobody against GFP. (**A**) Immunodetection was done by Western blot using anti-GFP and anti-RFP antibodies. (**B**) Quantification of the Gαo-Gβ1γ3 interaction for individual Gαo variants (*n* = 4–6). Bars are color-coded according to the involvement of Gαo mutants in the pathology developmental and epileptic encephalopathy-17 (DEE17; red) or neurodevelopmental disorder with involuntary movements (NEDIM; blue). (**C**) The combined Gβ1γ3 interaction of Gαo variants grouped in the DEE17 or NEDIM categories. (**D**–**F**) A scheme of the Gβ3γ9 displacement assay by BRET (**D**). Wild-type Gαo internally tagged with nano-luciferase (Gαo-NLuc) excites cpVenus (Ven) fused to Gγ9 in the Gβ3γ9 heterodimer. The ability of nontagged Gαo to displace Gβ3γ9 from Gαo-NLuc (reduction in the BRET signal) was quantified for wild-type Gαo, the encephalopathy mutants, and the GTPase-dead Q205L as control (*n* = 4–9) (**E**). The combined effect of the Gαo variants on Gβ3γ9 displacement sorted in the DEE17 or NEDIM group (**F**). (**G** and **H**) Scatterplots illustrating a strong positive correlation between Gβ3γ9 displacement and PM localization (**G**), and between disease onset and Gβ1γ3 co-IP (**H**) of Gαo mutants. Note the log scale in the *y* axis of **H**. Data represent mean ± SEM. Data in **B** and **E** were analyzed by 1-way ANOVA followed by Dunnett’s multiple-comparison test, in **C** and **F** by 2-tailed Mann-Whitney test, and in **G** and **H** by 2-tailed Spearman’s correlation test; rank correlation coefficients (*r_s_*) and *P* values are indicated. NS, not significant. *P < 0.05; **P < 0.01; ****P < 0.0001.

Our prior analyses of Gαo binding to Gβγ revealed different deviations from wild-type levels (10, 18). A systematic co-IP analysis of Gαo-GFP mutants coexpressed with mRFP-tagged Gβ1 and Gγ3 (25) uncovered varying perturbations of the Gαo-Gβγ interaction (Figure 3, A–C). As an independent confirmation of the co-IP studies, we employed a bioluminescence resonance energy transfer (BRET) displacement analysis (Figure 3D). Specifically, we measured the ability of nontagged Gαo variants to compete with the interaction between wild-type Gαo tagged with nano-luciferase (NLuc) and Gβ3γ9 with a Venus fusion (10, 27), revealing a perturbed Gαo-Gβγ pattern for the pathologic mutants similar to that seen in co-IPs (Figure 3, E and F, and Supplemental Figure 4B). These findings permit making the firm conclusion that decreased PM association strongly correlates with decreased interaction with Gβγ (Figure 3G and Supplemental Figure 4C), in agreement with the notion that Gα and Gβγ subunits require each other for a proper PM localization (28). Remarkably, a clear pattern emerges by both means to quantify Gαo-Gβγ interactions; mutations leading to DEE17 severely reduce Gβγ binding, while NEDIM mutations do not (Figure 3, C and F).

When looking for possible correlations between the clinical score and the parameters analyzed so far, we found poor/nonexisting correlations with Gαo expression levels (Supplemental Figure 2D), Golgi localization (Supplemental Figure 3C), or RGS19 interaction (Supplemental Figure 4A). In contrast, a sizable correlation existed between disease onset and Gαo PM association (Figure 2F) and Gβγ binding (Figure 3H and Supplemental Figure 4D). However, as the range of pathologic Gβγ interactions goes from better than wild-type (E246K) to worse than wild-type Gαo (G40R), the relative strength of the Gαo-Gβγ binding cannot serve as a simple biomarker to predict disease severity. Similarly, PM localization also cannot predict the clinical severity of the disease, as 2 mutations leading to DEE17 (S47G and G203R) showed near-normal membrane localization (Figure 2B and Supplemental Figure 3A).

### GPCR coupling of GNAO1 encephalopathy mutants.

**Figure 4:**
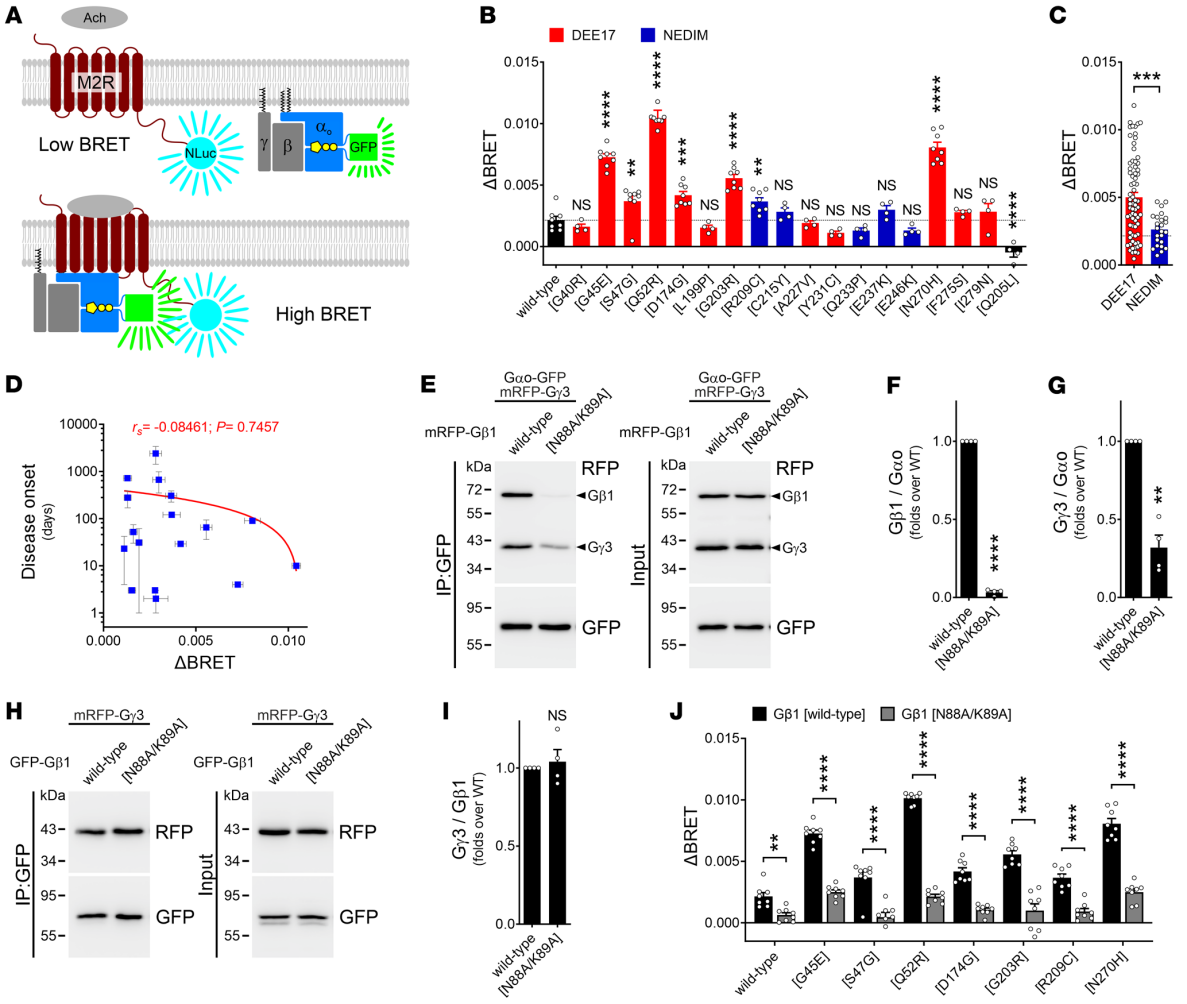
GPCR-coupling effects of *GNAO1* mutations. (**A**–**D**) The BRET-based M2 muscarinic acetylcholine receptor–coupling (M2R-coupling) assay (**A**). M2R tagged with nano-nuciferase (M2R-NLuc) excites the Gαo-GFP variants. The steady-state low BRET signal increased upon acetylcholine (Ach) treatment (ΔBRET), and the quantification for wild-type Gαo, mutants, and the GTPase-dead Q205L is shown (*n* = 4–8) (**B**). Data are color-coded according to the classification developmental and epileptic encephalopathy-17 (DEE17; red) and neurodevelopmental disorder with involuntary movements (NEDIM; blue). Effect of the Gαo mutants on M2R coupling pooled in the DEE17 or NEDIM group (**C**). A scatterplot showing a nonsignificant negative correlation between disease onset and M2R coupling by Gαo mutants (**D**). Note the log scale in the *y* axis of **D**. (**E**–**G**) N2a cells were cotransfected with Gαo-GFP, mRFP-Gγ3, and wild-type mRFP-Gβ1 or the N88A/K89A double mutant, and immunoprecipitation (IP) was done with a nanobody against GFP (**E**). Co-IP of Gβ1γ3 was analyzed by Western blot (WB) using antibodies against GFP and RFP. Quantification of the co-IP of Gβ1 (**F**) and Gγ3 (**G**) by Gαo (*n* = 4). (**H** and **I**) N2a cells were cotransfected with mRFP-Gγ3 and wild-type GFP-Gβ1 or N88A/K89A, and IP and WB were done as in **E**. Co-IP of wild-type Gγ3 by Gβ1 or mutant (*n* = 4) (**I**). (**J**) The effect of Gβ1 N88A/K89A on the M2R-coupling BRET assay for wild-type Gαo and selected mutants (*n* = 8). Data represent mean ± SEM. Data in **B** were analyzed by 1-way ANOVA followed by Dunnett’s multiple-comparisons test, in **C** by 2-tailed Mann-Whitney test, in **D** by 2-tailed Spearman’s correlation test (rank correlation coefficient [*r_s_*] and *P* value are indicated), in **F**, **G**, and **I** by 1-sample *t* test, and in **J** by 2-way ANOVA followed by Šídák’s multiple-comparison test. NS, not significant. **P < 0.01, ***P < 0.001, ****P < 0.0001.

As most of the Gαo mutants linked to DEE17 showed a poor PM expression and Gβγ association, we wondered whether these variants are capable of coupling with GPCRs. The tools currently used to analyze GPCR coupling of Gα subunits are based on BRET, but coupling is mainly determined indirectly by measuring Gα-Gβγ dissociation (27, 29–32). Direct engagement of Gα subunits with GPCRs has also been reported using BRET (33) or NLuc complementation (34). Thus, we determined the GPCR coupling of wild-type Gαo and pathogenic mutants by BRET, using an M2 muscarinic acetylcholine receptor C-terminally tagged with NLuc (M2R-NLuc) and the Gαo-GFP constructs (Figure 4A). Stimulation with acetylcholine induced, as expected (33), a modest increase in BRET over the basal signal in cells coexpressing M2R-NLuc together with wild-type Gαo, but not with the nonpathogenic Q205L mutant (Figure 4B). Strikingly, 7 pathogenic variants — G45R, S47G, Q52R, D174G, G203R, R209C, and N270H — showed significantly higher BRET signals upon stimulation, while the rest of the mutants behaved like wild-type Gαo. The mutations leading to DEE17 together presented a significantly higher BRET than NEDIM-linked mutants, which in turn displayed a nearly wild-type signal (Figure 4C). However, no significant correlation was calculated between disease onset and BRET signals (Figure 4D).

Among the group of the high-BRET responders, G45R, D174G, N270H, and our previously reported Q52R (18) displayed impairment in both PM localization and Gβγ association (Figure 2B and Figure 3, B and E). To confirm that the increase in the BRET signal by acetylcholine stimulation was indeed specific, we generated a Gβ1 mutant deficient in Gαo binding but capable of forming the Gβγ heterodimer. We speculated that expression of such a Gβ1 mutant would act as dominant negative over Gαo coupling to M2R by sequestering endogenous Gγ subunits. We thus introduced the N88A/K89A double point mutation into Gβ1 (35), and confirmed via co-IP its almost complete lack of Gαo interaction, without an effect on Gβ1γ3 formation (Figure 4, E–I). As predicted, Gαo co-IP of Gγ3 was strongly reduced in the presence of Gβ1 N88A/K89A (Figure 4, E and G), implying that it can indeed sequester Gγ subunits away from Gαo. We then performed the BRET assay in cells coexpressing M2R-NLuc with wild-type Gαo-GFP or the high-responder mutants, but now in the presence of Gβ1 N88A/K89A. Remarkably, all Gαo constructs showed a strong reduction in the BRET signal by the Gβ1 mutant (Figure 4J), confirming that Gαo G45R, Q52R, D174G, and N270H are capable of GPCR coupling despite their weak Gβγ association. The fact that several mutants displayed higher BRET signals than wild-type Gαo may suggest a deficient uncoupling from the stimulated GPCR, a property that might be relevant to the dominant nature of the *GNAO1* disease (9).

### Ric8 as neomorphic interaction partners of GNAO1 encephalopathy mutants.

**Figure 5:**
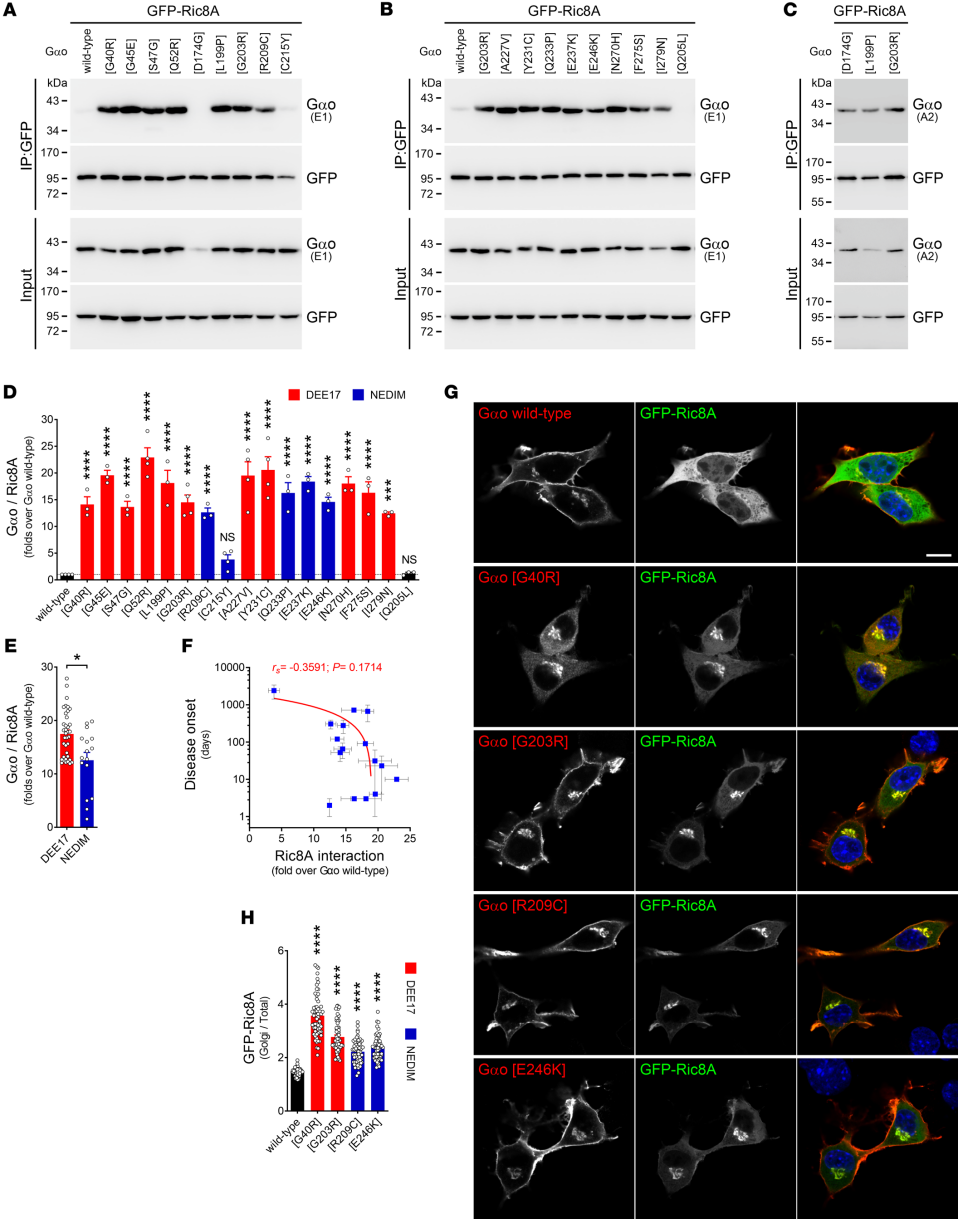
*GNAO1* mutants acquire a neomorphic interaction with Ric8A. (**A**–**C**) N2a cells were cotransfected with GFP-Ric8A and nontagged wild-type Gαo, encephalopathy mutants, and the GTPase-dead Q205L mutant as control. The immunoprecipitation (IP) of GFP-Ric8A was achieved with a nanobody against GFP and the interaction with Gαo variants was determined by Western blot (WB), using antibodies against GFP and against Gαo: clone E1 (**A** and **B**) or A2 (**C**). (**D**) Quantification of the co-IP of Gαo variants by Ric8A (*n* = 3–4). Data are color-coded according to their involvement in developmental and epileptic encephalopathy-17 (DEE17; red) or neurodevelopmental disorder with involuntary movements (NEDIM; blue). (**E**) The level of the Gαo-Ric8A interaction pooled according to DEE17 or NEDIM. (**F**) A scatterplot showing a nonsignificant negative correlation between disease onset and Ric8A interaction of Gαo mutants. Note the log scale in the *y* axis. (**G**) N2a cells coexpressing GFP-Ric8A and nontagged wild-type Gαo or selected encephalopathy mutants were immunostained against Gαo and stained with DAPI in blue for nuclei. (**H**) Quantification of the mean fluorescence intensity ratio of GFP-Ric8A at the Golgi versus total cell (*n* = 57–60). Scale bar: 10 μm. Data represent mean ± SEM. Data in **D** and **H** were analyzed by 1-way ANOVA followed by Dunnett’s multiple-comparison test, in **E** by 2-tailed Mann-Whitney test, and in **F** by 2-tailed Spearman’s correlation test; rank correlation coefficient (*r_s_*) and *P* value are indicated. NS, not significant. *P < 0.05; ***P < 0.001; ****P < 0.0001.

The defective properties of pathogenic Gαo mutants that we have described so far do not reveal a satisfactory biomarker to predict clinical severity, and do not identify the neomorphic function predicted by us (10). While searching for a neomorphic biomarker, we took into consideration the following observations: (i) several recombinant Gαo mutants are biochemically inactive (Supplemental Figure 1), (ii) most mutants have a decreased expression level (Supplemental Figure 2), and (iii) despite constitutive GTP loading, the mutants fail to interact with RGS19 (Figure 2, G and H). All these features hint at a potential folding problem, which prompted us to investigate Ric8A, which is a GEF and chaperone of Gαo and other Gα subunits (17, 36). To avoid any potential folding artifact due to the internal GFP fusion in Gαo, we used nontagged Gαo variants to analyze the Ric8A interactions. In accordance with Ric8A chaperone functions that presume only transient interactions with its clients, we found a very low capacity of GFP-tagged Ric8A to co-IP wild-type Gαo and Q205L (Figure 5, A and B). In contrast, all but one (C215Y) of the pathogenic Gαo mutants displayed a massive binding to Ric8A (Figure 5, A–D). Note that the D174G mutant is poorly recognized by the anti-Gαo antibody (Ab) used (clone E1; Figure 5A), but its neomorphic interaction with Ric8A was confirmed using another Ab (clone A2; Figure 5C). Interestingly, the mutants leading to DEE17 combined show a slightly, but significantly, more pronounced Ric8A interaction than the NEDIM mutants (Figure 5E), although no significant correlation could be seen between disease onset and Ric8A binding (Figure 5F).

**Figure 6:**
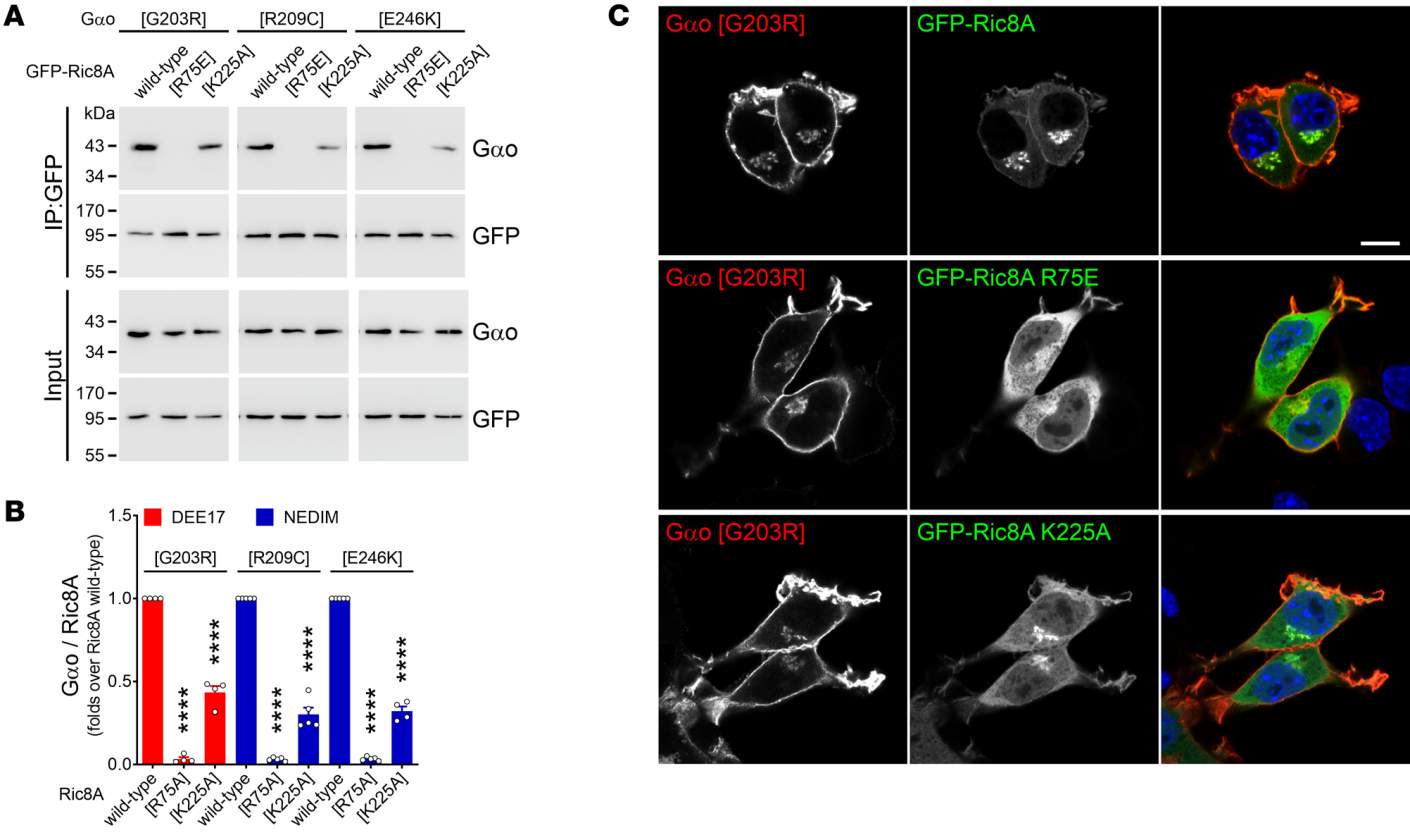
Chaperone-deficient mutants of Ric8A lose the neomorphic Gαo interaction. (**A** and **B**) N2a cells were cotransfected with GFP-Ric8A (wild-type or the chaperone-deficient mutants R75E and K225A) and nontagged Gαo mutants (G203R, R209C, or E246K). The immunoprecipitation (IP) of GFP-Ric8A variants was achieved with a nanobody against GFP, and the interaction with Gαo variants was determined by Western blot (WB) using antibodies against GFP and Gαo (**A**). Quantification of the co-IP of Gαo mutants by Ric8A constructs (*n* = 4–5) (**B**). Data are color-coded following the taxonomy developmental and epileptic encephalopathy-17 (DEE17; red) and neurodevelopmental disorder with involuntary movements (NEDIM; blue). (**C**) N2a cells coexpressing Gαo G203R and wild-type GFP-Ric8A, R75E, or K225A were immunostained against Gαo and stained in blue with DAPI to visualize the nuclei. Scale bar: 10 μm. Data represent mean ± SEM. Data in **B** were analyzed by 1-way ANOVA followed by Dunnett’s multiple-comparison test. ****P < 0.0001.

Ric8A is cytoplasmic, and this localization persists upon coexpression of wild-type Gαo or Q205L (Figure 5, G and H, and Supplemental Figure 5A). In contrast, every encephalopathy mutant induces a prominent neomorphic localization of GFP-Ric8A to the Golgi, and to a lower extent the PM (Figure 5, G and H, and Supplemental Figure 5A). Similar patterns were observed when HA-tagged Ric8A was cotransfected instead (Supplemental Figure 6A). The extent of mislocalization appeared similar across the mutants, with the sole exception of C215Y, with a weaker Golgi localization in accordance with its modest co-IP by Ric8A (Figure 5D and Supplemental Figure 5A). The Golgi relocalization of Ric8A is a direct consequence (and not prerequisite) of its Gαo binding, as incubation with the *N*-myristoylation inhibitor DDD85646 (25), which did not prevent the co-IP of Gαo mutants by Ric8A (Supplemental Figure 6, B and C), recovered the cytoplasmic localization of Ric8A (Supplemental Figure 6, D–F). To assess the specificity of this neomorphic interaction, we introduced point mutations into Ric8A known to abolish (R75E) or reduce (K225A) its chaperone activity (37). These Ric8A mutants showed a drastic decrease in binding to the Gαo variants (Figure 6, A and B), suggesting that pathogenic Gαo-Ric8A complexes are formed cotranslationally. As expected, these Ric8A constructs showed a clear reduction (K225A) or loss (R75E) of the Golgi relocalization by Gαo mutants (Figure 6C and Supplemental Figure 7, A and B).

We have previously described a *Drosophila* model of *GNAO1* encephalopathy that recapitulates clinical features seen in patients (10). The high degree of sequence identity and interchangeability of human and *Drosophila* Gαo (8) made us wonder whether the neomorphic Gαo-Ric8A interaction is evolutionarily conserved in *Drosophila*. Indeed, we found that *Drosophila* Gαo G203R interacts not only with *Drosophila* GFP-Ric8, but also with mammalian GFP-Ric8A (Supplemental Figure 8A). Similarly, human Gαo G203R was co-precipitated by *Drosophila* Ric8 (Supplemental Figure 8A). This result is somewhat surprising as, unlike the high degree of sequence identity between human and *Drosophila* Gαo, reaching 84% (8), the identity between the sole *Drosophila* Ric8 ortholog and mammalian Ric8 is quite limited: 35% to Ric8A and 33% to Ric8B, with both mammalian Ric8 proteins being 47% identical (Supplemental Figure 8B).

**Figure 7:**
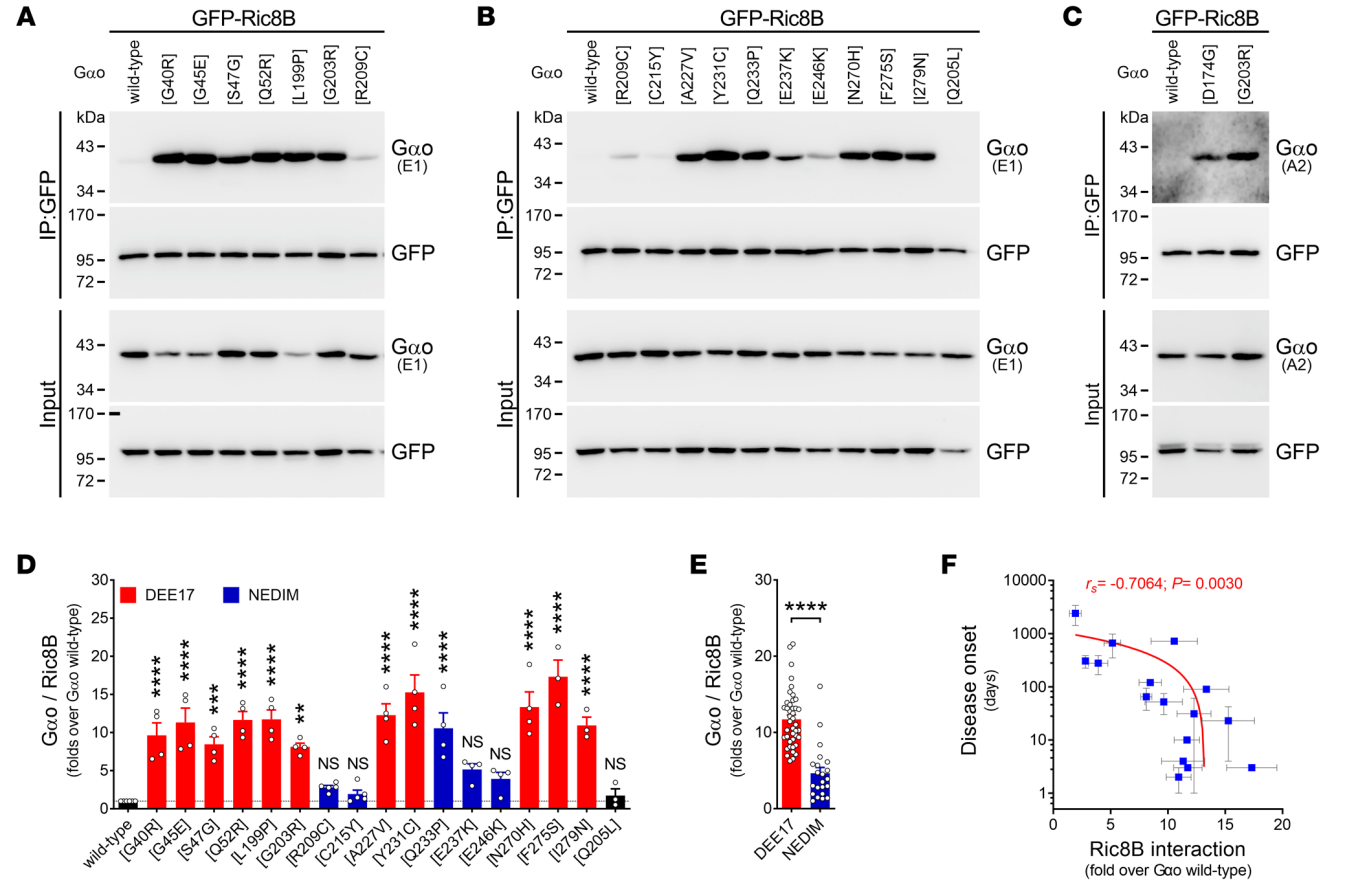
Ric8B neomorphic interaction with *GNAO1* mutants. (**A**–**C**) N2a cells were cotransfected with GFP-Ric8B and nontagged wild-type Gαo, encephalopathy mutants, and Q205L as control. The immunoprecipitation (IP) of GFP-Ric8B was achieved with a nanobody against GFP and the interaction with Gαo variants was determined by Western blot (WB), using antibodies against GFP and Gαo clone E1 (**A** and **B**) or A2 (**C**). (**D**) Quantification of Gαo pulled down by Ric8B (*n* = 3–5). Data are color-coded according to the involvement in developmental and epileptic encephalopathy-17 (DEE17; red) or neurodevelopmental disorder with involuntary movements (NEDIM; blue). (**E**) Level of the Gαo-Ric8B neomorphic interactions according to DEE17 or NEDIM. (**F**) A scatterplot illustrating a significant negative correlation between disease onset and the level of Gαo coprecipitated by Ric8B. Note the log scale in the *y* axis. Data represent mean ± SEM. Data in **D** were analyzed by 1-way ANOVA followed by Dunnett’s multiple-comparison test, in **E** by 2-tailed Mann-Whitney test, and in **F** by 2-tailed Spearman’s correlation test; rank correlation coefficient (*r_s_*) and *P* value are indicated. NS, not significant. **P < 0.01, ***P < 0.001, ****P < 0.0001.

**Figure 8:**
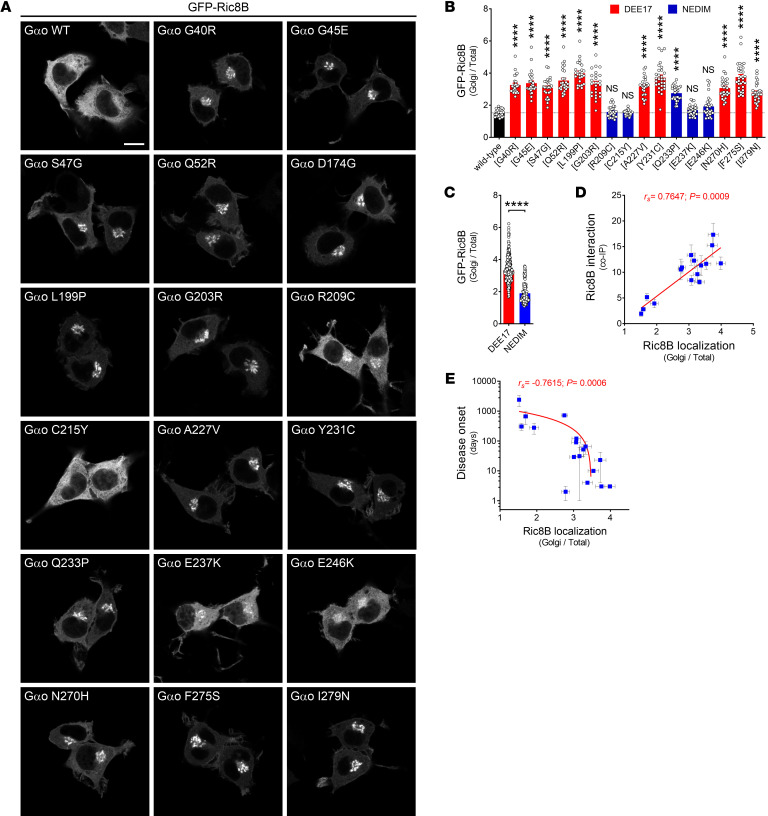
Severe *GNAO1* mutations induced a Golgi relocalization of Ric8B. (**A**) Representative images of the localization of GFP-Ric8B in N2a cells coexpressing wild-type Gαo or mutants (Gαo immunostaining not shown). Scale bar: 10 μm. (**B**) Quantification of the mean fluorescence intensity ratio of GFP-Ric8B at the Golgi versus total cell (*n* = 25–30). Bars are color-coded according to the Gαo involvement in developmental and epileptic encephalopathy-17 (DEE17; red) or neurodevelopmental disorder with involuntary movements (NEDIM; blue). (**C**) Relative Golgi localization of Ric8B pooled according to DEE17 and NEDIM. (**D** and **E**) Scatterplots showing the significant correlation between Ric8B Golgi localization and Ric8B interaction with Gαo mutants (**D**) or disease onset (**E**). Note the log scale in the *y* axis in **E**. Data represent mean ± SEM. Data in **B** were analyzed by 1-way ANOVA followed by Dunnett’s multiple-comparison test, in **C** by 2-tailed Mann-Whitney test, and in **D** and **E** by 2-tailed Spearman’s correlation test; rank correlation coefficient (*r_s_*) and *P* value are indicated. NS, not significant. ****P < 0.0001.

These findings prompted us to question whether the Gαo encephalopathy mutants can additionally gain a neomorphic interaction with Ric8B — the isoform “foreign” for Gαo, but specific instead for Gαs and Gαolf (17). Surprisingly, we found that all Gαo mutants leading to DEE17 were strongly pulled down by GFP-Ric8B, while Ric8B binding by the NEDIM group (with the exception of Q233P) was less pronounced (Figure 7, A–E). Remarkably, we found a strong correlation between disease onset and Ric8B interaction (Figure 7F). Similarly to Ric8A, we also observed a strong cytoplasm-to-Golgi mislocalization of Ric8B, but only in a subset of mutants (Figure 8A). Quantification revealed that the relative Golgi localization of GFP-Ric8B was significantly increased for all DEE17 variants and for Q233P (Figure 8B), and the DEE17 mutants combined showed a much higher Golgi presence of Ric8B than their NEDIM counterparts (Figure 8C). As expected, the level of Ric8B localization at the Golgi strongly correlated with both Gαo-Ric8B co-IP and disease onset (Figure 8, D and E).

Altogether, the strength of the neomorphic Ric8B interaction provides the best predictive value for the clinical manifestations of *GNAO1* encephalopathy mutations.

### A global effect of GNAO1 encephalopathy mutants over Gα subunits.

**Figure 9:**
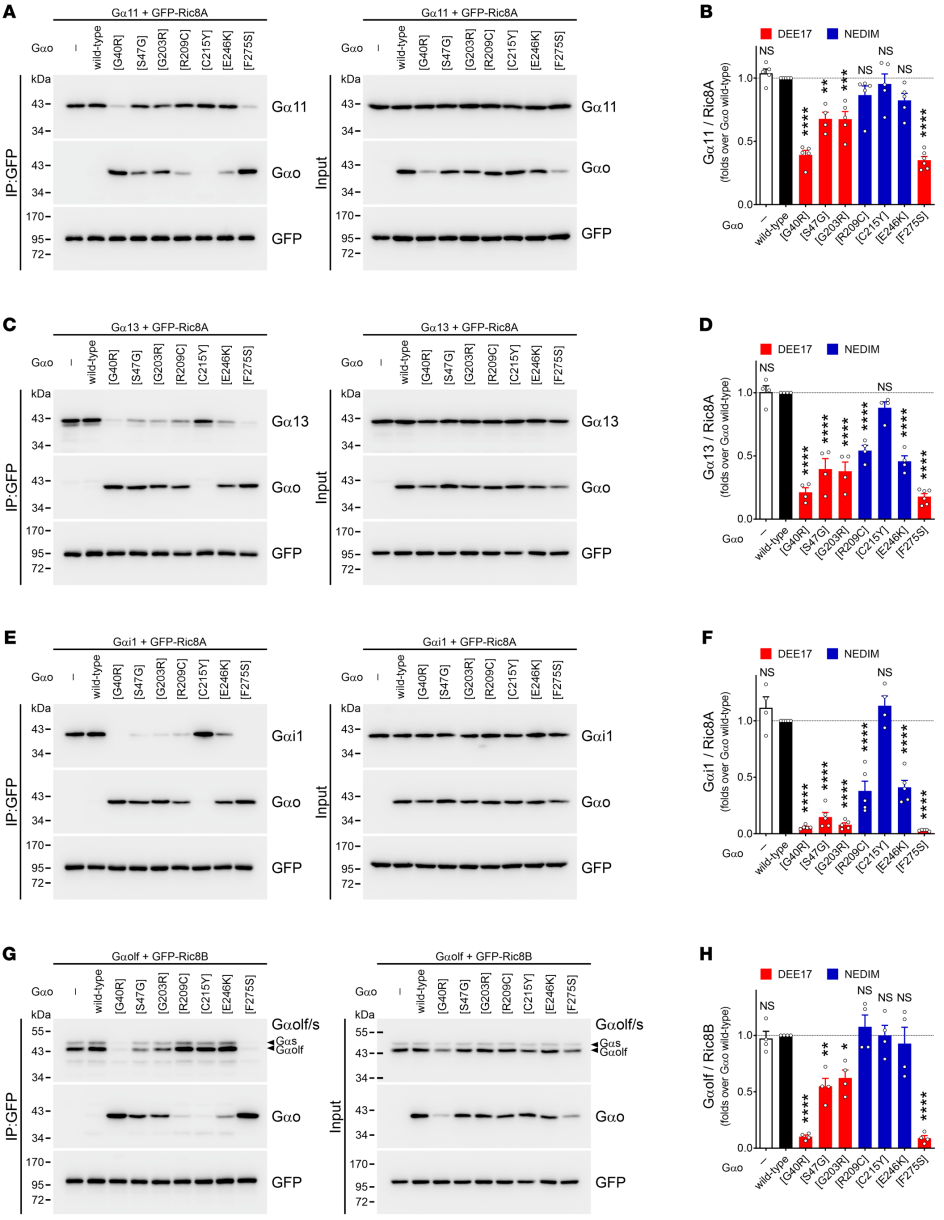
*GNAO1* mutants interfere with Ric8 binding to Gα subunits. (**A**–**H**) HEK293T cells were cotransfected with GFP-tagged Ric8A (**A**, **C**, and **E**) or Ric8B (**G**), the nontagged Gα11 (**A**), Gα13 (**C**), Gαi1 (**E**), or Gαolf (**G**), and wild-type Gαo, encephalopathy mutants, or empty plasmid (–). The immunoprecipitation (IP) of GFP-Ric8A/B was done using a nanobody against GFP and the interaction with the Gα subunits was determined by Western blot (WB), using antibodies against GFP, Gαo, and Gα11, Gα13, Gαi1, or Gαolf/Gαs. Quantification of the interaction between Ric8A/B with the indicated Gα subunits (*n* = 4–6) (**B**, **D**, **F**, and **H**). Data are color-coded according to the involvement in developmental and epileptic encephalopathy-17 (DEE17; red) or neurodevelopmental disorder with involuntary movements (NEDIM; blue). Data represent mean ± SEM. Statistical analysis in **B**, **D**, **F**, and **H** was done by 1-way ANOVA followed by Dunnett’s multiple-comparison test. NS, not significant. *P < 0.05, **P < 0.01, ***P < 0.001, ****P < 0.0001.

Due to the strong interactions with Ric8A and Ric8B proteins gained by the pathogenic Gαo variants, we next wondered whether this may affect the normal GEF and chaperone activities of Ric8A/B toward the other Gα subunits (37–39). We first immunoprecipitated GFP-tagged Ric8A and Ric8B from HEK293T cells coexpressing at least one member of each Gα subfamily in order to assess their interactions. Interestingly, unlike the Gαo-Ric8A binding that was rather weak for wild-type Gαo, we observed that Gα11, Gα13, Gαi1, and Gαq were efficiently pulled down by Ric8A, and Gαolf and Gαs by Ric8B (Supplemental Figure 9, A–F). Both members of the Gαq subfamily (Gα11 and Gαq) co-precipitated with Ric8B as well, in agreement with a previous report (40). Next, we repeated the co-IPs for the main Gα-Ric8 pairs, but in the presence of wild-type Gαo or selected pathogenic mutants. From the DEE17-linked mutants, we chose G40R and F275S, which present a very poor PM and Gβγ association, and S47G and G203R with near-normal PM localization and Gβγ interaction (Figure 2B and Figure 3, B and E). We additionally picked the recurrent NEDIM mutations R209C and E246K, and the outlier C215Y. Noteworthily, all tested DEE17 variants significantly impaired Ric8A interactions with Gα11, Gα13, and Gαi1 following a clear pattern; G40R and F275S showed the strongest effect, followed by S47G and G203R (Figure 9, A–F). The NEDIM variants showed mixed results, with R209C and E246K clearly impairing Ric8A interactions with Gα13 and Gαi1 but not Gα11, and C215Y presenting no significant effects (Figure 9, A–F). The Gαq-Ric8A interaction was affected by Gαo mutants, following a pattern similar to that of Gα11 (Supplemental Figure 9G). Even more remarkable was the effect of DEE17 variants on the Gαolf-Ric8B interaction, with G40R and F275S almost completely outcompeting Gαolf (Figure 9, G and H). Conversely, all NEDIM variants did not significantly impair Gαolf binding to Ric8B. The Gαs-Ric8B interaction was mildly affected, but only by G40R and F275S (Supplemental Figure 9H). We asked next whether the competition for Ric8 binding by the Gαo mutants interferes with the subcellular localization of another Gα subunit. However, a Gαq-GFP construct was still present at the PM in cells coexpressing Gαo G40R, G203R, and E246K (Supplemental Figure 10A), in agreement with the notion that Ric8 loss of function does not block PM targeting of Gα subunits (17).

**Figure 10:**
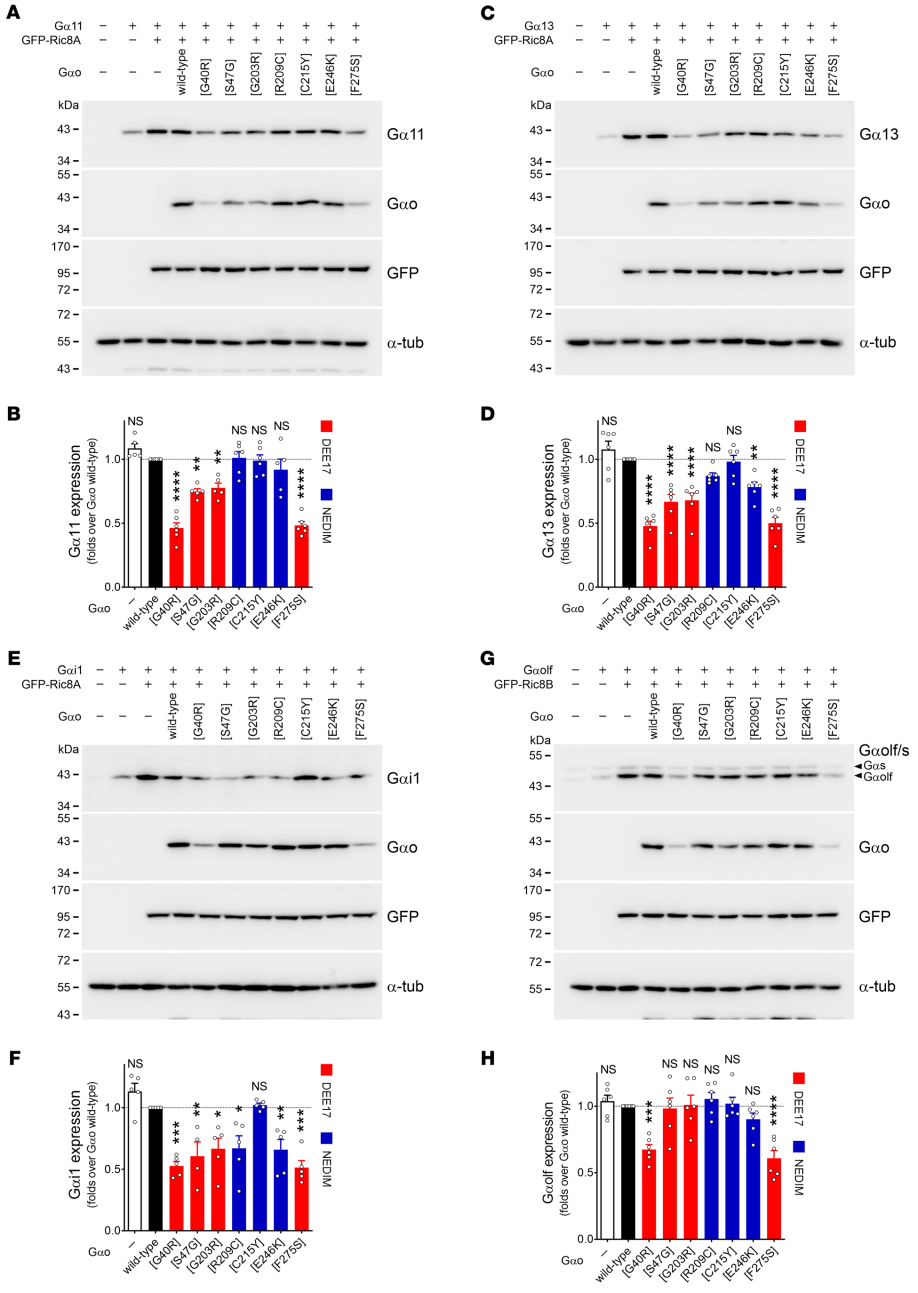
The chaperone activity of Ric8 is affected by *GNAO1* mutants. (**A**–**F**) HEK293T Ric8A-KO cells were cotransfected with GFP-Ric8A, wild-type Gαo, encephalopathy mutants, or empty plasmid (–), and Gα11 (**A**), Gα13 (**C**), or Gαi1 (**E**). Samples were analyzed by Western blot (WB) using antibodies against GFP, Gαo, Gα11, Gα13, Gαi1, and α-tubulin (α-tub) as loading control. The expression levels of Gα11 (**B**), Gα13 (**D**), or Gαi1 (**F**) were normalized to GFP-Ric8A signal (*n* = 5–6). Data are color-coded following the association with developmental and epileptic encephalopathy-17 (DEE17; red) or neurodevelopmental disorder with involuntary movements (NEDIM; blue). (**G** and **H**) HEK293T cells were cotransfected with GFP-Ric8B, Gαolf, and wild-type Gαo, encephalopathy mutants, or empty plasmid (–). Samples were analyzed and quantified as in **A**–**F**, and an antibody against Gαolf was used for immunodetection (*n* = 6). Data represent mean ± SEM. Statistical analysis in **B**, **D**, **F**, and **H** was done by 1-way ANOVA followed by Dunnett’s multiple-comparison test. NS, not significant. *P < 0.05, **P < 0.01, ***P < 0.001, ****P < 0.0001.

Thus, we conclude that pathogenic Gαo variants significantly outcompete Ric8A/B from their other cognate Gα subunits. We next speculated that such out-competition could affect the Ric8 chaperone activity toward these Gα subunits, reflected by their reduced levels. To determine de novo expression of Gα subunits, we generated a CRISPR/Cas9-based Ric8A-knockout (Ric8A-KO) HEK293T cell line. Similarly to prior observations (17), KO of Ric8A led to reduced levels of Gα11, Gα13, and Gαi1 in this cell line (Supplemental Figure 10B). We then reintroduced Ric8A (GFP-Ric8A) together with individual Gα subunits and Gαo variants. Notably, the expression of Gα11, Gα13, and Gαi1 was strongly reduced (~50%) by the coexpression of the DEE17 mutants G40R and F275S, and to a lesser extent (30%–40%) by S47G and G203R (Figure 10, A–F). The NEDIM variants induced weaker defects, with E246K significantly impairing Gα13 and Gαi1 but not Gα11, R209C affecting Gαi1, and C215Y showing no effect (Figure 10, A–F). A defective Ric8B chaperone activity toward Gαolf was noticeable by its lower expression level upon Gαo G40R and F275S coexpression in the co-IP analysis (Figure 9G), and a formal quantification revealed an approximately 35% reduction in Gαolf expression by these DEE17 mutants (Figure 10, G and H).

Overall, the neomorphic Gαo-Ric8 interaction seen for the pathogenic variants may lie at the core of the disease dominance, affecting not only Gαo signaling, but also imbalancing the entire neuronal GPCR signaling network.

## Discussion

Recent advances in next-generation whole-exome/genome sequencing have uncovered a plethora of *GNAO1* mutations associated with a rare, yet devastating, pediatric encephalopathy. The number of patients has steadily increased since the first reported cases in 2013 (4). The understanding of the molecular etiology underlying the pathology, however, remains uncertain despite some current progress (4, 9–11, 14, 15, 26). In our previous study, we hypothesized that *GNAO1* encephalopathy mutations are neomorphic in nature (10), in the classical Muller categorization of genetic mutations. Citing from Muller’s seminal paper (41), neomorph represents a “change in the nature of the gene at the original locus, giving an effect not produced, or at least not produced to an appreciable extent, by the original normal gene.” In cancer, many oncogenic mutations in different genes, previously considered gain- or loss-of-function mutants, now emerge as neomorphs (42). Here, through a massive characterization of *GNAO1* mutations, we uncovered a neomorphic feature shared by all Gαo encephalopathy mutants: a strong gain of interaction with Ric8A and, even more surprisingly, with Ric8B.

Initially described as GEFs (36), Ric8 proteins have subsequently emerged as mandatory chaperones for Gα subunits (17), with Ric8A responsible for the Gαi/Gαo, Gαq, and Gα12/Gα13 subfamilies, and Ric8B solely for Gαs/Gαolf. Ric8 interactions with Gα subunits are highly specific, with Ric8B being unable to engage members of the Gαi/Gαo class (36, 43). As the main determinant for Ric8B specificity resides in the extended C-terminal α5 helix of Gαs/Gαolf, which is shorter in Gαi/Gαo members (39), it is tempting to speculate that pathogenic *GNAO1* mutations alter Gαo structure in ways that differentially accommodate its α5 helix for Ric8B. Given the effect of the pathogenic Gαo variants over the binding and chaperone function of Ric8 for several Gα subunits, the neomorphic Gαo-Ric8 interaction may lie at the core of the disease dominance, affecting not only Gαo signaling, but also imbalancing the entire neuronal GPCR signaling network. This neomorphic property might explain why several Gαo variants, despite been poorly expressed, lead to the severe DEE17 phenotype instead of the milder dystonic phenotype recently linked to *GNAO1* haploinsufficiency (19, 44, 45).

Our findings may go beyond the pediatric neurological disorders caused by mutations in *GNAO1*. Indeed, missense mutations in genes encoding other Gα subunits, often in the same amino acids as found mutated in *GNAO1* encephalopathy, underlie a broad variety of genetic diseases. One example is Gαi1, a close relative of Gαo, also showing prominent CNS expression; missense mutations in G40, G45, Q52, or D173 of Gαi1 (equivalent to the mutations found in *GNAO1* encephalopathy) cause dominant infantile neurological disorders, with variable degrees of developmental delay, seizures, and hypotonia (18, 46). Dominant mutations in Gαs in position R231 (R209 in Gαo) or E259 (E237 in Gαo) cause Albright’s hereditary osteodystrophy, with skeletal and developmental abnormalities including brachydactyly, short stature, obesity, and mental deficits (47). Gα11 has been found to carry dominant point mutations in autosomal dominant hypocalcemia and in hypocalciuric hypercalcemia, disorders of mineral homeostasis, including mutations in positions R181 and S211 (R177 and S207 mutated in *GNAO1* encephalopathy) (48). These dominant mutations scattered across Gα sequences are distinct from the classical activating mutations. We hypothesize that the common mechanism, involving neomorphic engagement of Ric8 chaperones and imbalancing the whole network of GPCR/G protein signaling pathways, is at the core of these diseases, with the exact disease manifestation (neurologic, developmental, metabolic, etc.) being dependent on the tissue where the affected Gα subunit is abundantly expressed. In this perspective, targeting the neomorphic Gα-Ric8 interactions may emerge as an attractive approach for future drug discovery efforts aiming at a broad range of G protein–linked diseases.

Our study provides a systematic investigation of a large panel of pathogenic Gαo mutants across several biochemical and cellular assays, revealing that the mutants tend to bind GTP faster but lose GTP hydrolysis, lose interaction with RGS19, display varying deficiencies in PM localization and Gβγ binding, and gain a neomorphic Ric8 interaction. However, Gαo C215Y stands out from the group, as it shows an increased Gβγ interaction, normal subcellular localization, and a moderate binding exclusively to Ric8A, but not Ric8B. C215Y also loses RGS19 binding, although this might reflect its nucleotide-binding state state in cells, as it is the only Gαo mutant predicted to have a higher GDP loading than wild-type. Accordingly, the C215Y variant, as the T241_N242insPQ (c.724–8G > A) mutant recently characterized by us (49), falls into the milder end of the spectrum of *GNAO1* encephalopathy with adolescent/adult onset (19). Along the same line, we recently showed that 2 *GNAO1* mutations affecting the N-terminus of Gαo — L13P and L23P — lose Gβγ binding without acquiring the neomorphic Ric8 interaction, in agreement with their association with a mild parkinsonism phenotype (50).

During the revision of this study, 2 separate publications appeared describing biochemical and/or cellular properties of several pathogenic Gαo variants (51, 52). While the study by Knight et al. does not address the genotype-phenotype correlation of *GNAO1* mutations (52), Dominguez-Carral et al. identified Gβγ binding as the best predictor for clinical severity (51). Although we also found a correlation for the defects in Gβγ interaction, we conclude that it cannot serve as an efficient predictor of disease severity, and in fact loss of heterotrimer formation completely fails to agree with severe clinical manifestations in some cases (50). In our work, we tested such characteristics of pathogenic Gαo variants as (i) expression levels, (ii) ability to bind and hydrolyze guanine nucleotides, (iii) intracellular localization, (iv) binding to physiological interaction partners (RGS19 and Gβγ), (v) GPCR coupling, and (vi) neomorphic interactions with Ric8A and Ric8B proteins. While important features could be found to correlate with the disease severity and manifestations (such as the loss of PM localization being a good predictor of the epileptic phenotype), the neomorphic interactions emerge as the most informative. Indeed, out of multiple deficiencies of pathogenic Gαo variants, the interaction with Ric8B appears not only as the most unexpected neomorphic feature of the disease, but also as the best predictor of its severity. *GNAO1* encephalopathy is a recently discovered disorder, and most known patients are infants with unclear prognosis. The molecular outcome of many *GNAO1* mutations remains unknown, as sequencing continues to identify more patients, often with novel mutations. With this background, a simple biomarker of the disease severity is in high demand. Our work demonstrates the Gαo-Ric8B interaction as such a biomarker, with the strength of the interaction correlating with disease severity. A simple test measuring Ric8 interactions with new variants may become the routine way for medical geneticists and pediatric neurologists to evaluate the expected disease severity, long-term prognosis, and treatment.

In summary, our work sheds light on the molecular etiology of *GNAO1* encephalopathy, identifies the neomorphic intermediates of the disease dominance, provides a much-needed biomarker for assessment of disease severity, and might pave the way for future drug discovery for this disorder. The realization that the mutations underlying *GNAO1* encephalopathy are neomorphic (as opposed to the more traditional loss- or gain-of-function dichotomy) suggests reconsidering the genetic basis of many other genetic diseases linked to mutations in genes encoding Gα subunits, but also in unrelated genes.

## Methods

### Sex as a biological variable.

In this study, sex was not considered as a biological variable.

### Abs and reagents.

Primary Abs for immunofluorescence (IF) and Western blots (WBs): monoclonal Abs (mAbs) anti-Gαo (clone A2, sc-13532; WB: 1:50, IF: 1:50), anti-Gαo (clone E1, sc-393874; WB: 1:250), anti-mRFP/DsRed2 (sc-101526; WB: 1:250), anti-Gα11 (sc-390382; WB: 1:100), anti-Gαi1 (sc-13533; WB: 1:50), anti-Gαs/Gαolf (sc-377435; WB: 1:100), and anti-Gβ (recognizes Gβ1–Gβ4; sc-166123; WB: 1:100) were from Santa Cruz Biotechnology; mAb anti-His_6_ (34650; WB: 1:1000) from Qiagen, mAb anti-GM130 (610823; IF: 1:500) from BD Biosciences, mAb anti–α-tubulin (T6199; WB: 1:2000) from Sigma-Aldrich, and mAbs anti-Gα13 (67188-1-Ig; WB: 1:1000) and anti-Ric8A (66625-1-Ig; WB: 1:1000) were from Proteintech. Polyclonal antibody (pAb) anti-GFP (GTX113617; WB: 1:2000) was from GeneTex, pAb against HA tag (ab9110; IF: 1:500) was from Abcam, and pAb anti-Gαq (13927-1-AP; WB: 1:1000) was from Proteintech. The detailes for the pAb against *Drosophila* Gαo (WB: 1:2000) were previously published (53). All secondary Abs for IF and WBs were from Jackson ImmunoResearch: anti–mouse Cy3-conjugated (115-165-146; IF: 1:500), anti-rabbit Alexa Fluor 488–conjugated (111-545-144; IF: 1:500), anti-mouse HRP-conjugated (115-035-146; WB: 1:5000), and anti-rabbit HRP-conjugated (111-035-144; WB: 1:5000). DAPI (Sigma-Aldrich, 32670), VECTASHIELD Mounting Medium (Vector Laboratories, H-1400), Glutathione Sepharose 4B beads (Cytiva, 17075601), and DDD85646 (Cayman Chemical, 13839) were also used.

### Plasmids and molecular cloning.

The plasmids encoding nontagged Gαo, His_6_-tagged Gαo, and Gαo-GFP (GFP insertion between residues G92 and I93) for the wild-type and mutants (Q52R, G203R, Q205L, R209C, and E246K), His_6_-RGS19, GFP-Gβ1, mRFP-Gβ1, HA-Gγ3, mRFP-Gγ3, and nontagged *Drosophila* Gαo were previously described (3, 10, 18, 22, 23). Additional Gαo mutants G40R, G45E, S47G, D174G, L199P, C215Y, A227V, Y231C, Q233P, E237K, N270H, F275S, and I279N, and *Drosophila* Gαo G203R were obtained by point mutagenesis using oligonucleotide primers (all primers used in this study are listed in Supplemental Table 2). The Gβ1 N88A/K89A mutant was similarly obtained by point mutagenesis. pcDNA3.1(+) plasmids encoding the nontagged Gα11 (GNA1100000), Gα13 (GNA1300001), Gαi1 (GNAI100000), Gαolf (GNA0L00000), Gαq (GNA0Q00000), and Gαs (GNA0SL000) were obtained from the cDNA Resource Center. The Gαq construct with an internal GFP fusion (54) was from Addgene (plasmid 66080). The M2 muscarinic receptor sequence (MAR0200000; cDNA Resource Center) was amplified by PCR and cloned in frame into the EcoRI/AgeI sites of the pNLuc-N1 plasmid (provided by Nevin A. Lambert, Augusta University, Augusta, Georgia, USA). To generate GFP-Ric8A, the Ric8A sequence was amplified by PCR from the HA-Ric8A plasmid (55) (provided by Yijuang Chern, Academia Sinica, Taipei, Taiwan) and cloned in frame into the SalI/PspOMI sites of pEGFP-C3 (Clontech). The R75E and K225A mutations were introduced into GFP-Ric8A by point mutagenesis. Ric8B was PCR amplified from pB-Ric8B (56) (Addgene plasmid 129457) and cloned in frame into the XhoI/EcoRI sites of pEGFP-C1 (Clontech), producing the GFP-Ric8B construct. To clone a GFP fusion of dRic8 (*Drosophila*), the dRic8 sequence was PCR amplified from pMT-GFP-dRic8 (57) (provided by Stephen Rogers, University of North Carolina at Chapel Hill, Chapel Hill, North Carolina, USA) and inserted in frame into the BsrGI/PspOMI sites of pEGFP-C1.

### Recombinant protein purification.

His_6_-tagged Gαo proteins were expressed in *E*. *coli* Rosettagami (Novagen, 71351), as previously described (10). Briefly, transformed bacteria were grown in baffled flasks at 37°C to an OD_600_ of 0.6, cooled down to 18°C for at least 30 minutes before induction with 1 mM isopropyl-β-D-thiogalactopyranoside, and were additionally grown overnight at 18°C. Bacteria were harvested by centrifugation at 3,500*g* and 4°C and resuspended in TBS (20 mM Tris-HCl [pH 7.5] and 150 mM NaCl) supplemented with 1 mM PMSF and 30 mM imidazole (all Sigma-Aldrich). Cells were disrupted with a OneShot high-pressure cell press disruptor (CONSTANT Systems) at 0.7 kbar and extracts were cleared by centrifugation at 15,000*g* for 15 minutes at 4°C. Supernatants were incubated with Ni-NTA Agarose beads (QIAGEN) overnight in a rotary shaker at 4°C. Beads were washed twice with 10 volumes of wash buffer (TBS supplemented with 10 mM imidazole) and bound proteins were GDP loaded in TBS supplemented with 3% glycerol, 10 mM MgCl_2_, 0.1 mM DTT, and 200 μM GDP. Beads were washed 2 more times with at least 10 volumes of ice-cold wash buffer and finally eluted with TBS containing 300 mM imidazole. Imidazole was removed by buffer exchange to TBS using Vivaspin Centrifugal concentrators. Protein concentration was measured using the Bradford assay, and the purity was analyzed using SDS-PAGE followed by Coomassie staining.

### GTP binding and hydrolysis assay.

The GTP binding and hydrolysis assay using BODIPY-GTP or BODIPY-GTPγS (both from Invitrogen) was performed as described previously (3). Briefly, His_6_-Gαo recombinant proteins were diluted to 1 μM in reaction buffer (TBS supplemented with 10 mM MgCl_2_ and a 0.5% BSA). The mixture was then pipetted into black 384-well plates (Greiner), and BODIPY-GTP or BODIPY-GTPγS was added into the wells to 1 μM final concentration. Fluorescence measurements were performed at 28°C in a Tecan Infinite M200 PRO plate reader with excitation at 485 nm and emission at 530 nm. In all cases, the fluorescent ligand was injected into the wells as half of the final volume of the reaction mixture using the injector system of the plate reader. The GTP binding and hydrolysis data of wild-type Gαo were fit to obtain the *k*_bind_ and *k*_hydr_ rate constants as previously described (3), setting the end point as the baseline. For Gαo mutants with strongly impaired GTP hydrolysis, the maximal duration of the assay was not sufficient to reach complete hydrolysis and thus the *k*_hydr_ was extrapolated using the available BODIPY-GTP curve, with the initial fluorescence value as a projected baseline.

### Modeling GαoGTP/GαoGDP ratios.

Considering a ca. 10-fold excess of free GTP relative to GDP in cells, setting the cellular concentration of Gαo at 10 μM, and considering a ca. 100-fold excess of guanine nucleotides over the G proteins (24), in the absence of GEFs, GAPs, or GDIs the ratio of the GTP-loaded Gαo to that in the GDP-bound state is determined by the simple equations:

[Gαo*^GDP^*]/*dt* = *k*_hydr_[Gαo*^GTP^*] – *k*_bind_[Gαo*^GDP^*] Eq. 1

[Gαo*^GTP^*]/*dt* = *k*_bind_[Gαo*^GDP^*] – *k*_hydr_[Gαo*^GTP^*] Eq. 2

Knowing the *k*_bind_ and *k*_hydr_ values for wild-type and mutant Gαo, these differential equations were numerically solved using power law analysis and simulation (PLAS) software (https://github.com/SMRUCC/PLAS.NET) as described previously (24). Resulting equilibrium concentrations of Gαo^GTP^ and Gαo^GDP^ provided the [Gαo^GTP^]/[Gαo^GDP^] ratios, shown in Supplemental Figure 1D.

### Cell lines and culture conditions.

Mouse N2a (CCL-131, ATCC) cells were maintained in MEM (Thermo Fisher Scientific), supplemented with 10% FCS, 2 mM L-glutamine, 1 mM pyruvate, and 1% penicillin-streptomycin at 37°C and 5% CO_2_. Human HEK293T (CRL-3216, ATCC) cells were grown in DMEM (Thermo Fisher Scientific), supplemented as above and under the same culture conditions. To generate HEK293T Ric8A-KO cells, we used the CRISPR/Cas9 gene editing system based on lentiGuide-Puro and lentiCas9-EGFP vectors (58). The following guide RNAs (gRNAs) were designed using the CHOPCHOP tool (https://chopchop.cbu.uib.no/) and cloned into the lentiGuide-Puro vector: Ric8A_f 5′-CCTAGTGGTGAAGCTCACAGAG-3′ and Ric8A_r 5′-GGTGATGTTGAAGAGCACTTTG-3′. HEK293T cells were transfected and selected under 10 μg/mL puromycin. Single clones were split and the KO of Ric8A was screened by WB and additionally validated by PCR amplification followed by Sanger sequencing. All vector transfections were carried out with X-tremeGENE HP (XTGHP-RO, Roche) or FuGENE HD (E2311, Promega) according to the manufacturer’s instructions.

### IF and microscopy.

N2a (1.5 × 10^5^ cells/well) and HEK293T (1 × 10^5^ cells/well) were seeded on culture plates, and 24 hours later were transfected for 7 hours, trypsinized, and seeded on poly-L-lysine–coated coverslips in complete MEM or DMEM for an additional 15–17 hours before fixation. When indicated, cells were seeded in complete MEM supplemented with 10 μM DDD85646 or DMSO as control. Cells were fixed with 4% paraformaldehyde in PBS for 20 minutes, permeabilized for 1 minute using ice-cold PBS supplemented with 0.1% Triton X-100, and blocked for 30 minutes with PBS supplemented with 1% BSA. Cells were then incubated with the primary Abs in blocking buffer for 2 hours at room temperature, washed, incubated with secondary Abs and DAPI also in blocking buffer for 2 hours at room temperature, and coverslips were finally mounted with VECTASHIELD on microscope slides. Cells were recorded with a Plan-Apochromat ×63/1.4 oil objective on an LSM 800 confocal microscope using ZEN 2.3 software (all Zeiss). When required, mean fluorescence intensity was determined from confocal images using ImageJ v1.54f (NIH). Images were not recorded using the same confocal settings; therefore, ratios of fluorescence values, such as Golgi fluorescence versus total fluorescence or PM versus total, were used for quantifications as previously validated (25). All images were finally edited using ZEN lite 3.3 (Zeiss) and CorelDRAW 2020 (Corel).

### PM and Golgi localization.

N2a cells were transfected with 0.5 μg of wild-type Gαo-GFP or mutant plasmids, immunostained against GM130 to visualize the Golgi apparatus, and stained with DAPI for nuclei (as indicated above). For Ric8A/B localization studies, N2a cells were cotransfected with nontagged Gαo (0.2 μg) and GFP-Ric8A, HA-Ric8A, or GFP-Ric8B (0.4 μg), immunostained against Gαo and when needed against the HA tag, and stained with DAPI (not shown for all images). To avoid interference due to cell variability in expression of the constructs, mean fluorescence intensity was measured at the Golgi region as well as at the total cell area, and ratios were used to determine their relative Golgi accumulation. Likewise, mean fluorescence intensity was determined at an unbroken PM region without membrane protrusions, and the ratio over total cell fluorescence was used to define relative PM content for each Gαo-GFP construct.

### Biochemical analyses.

For Gαo expression analysis, N2a cells were seeded on culture plates (1.5 × 10^5^ cells/well) and 24 hours later were transfected with 0.5 μg of wild-type Gαo-GFP or mutants. After an additional 24 hours, cells were harvested with Accutase (Thermo Fisher Scientific), lysed in Laemmli buffer with sonication, heated at 95°C for 5 minutes, and finally analyzed by SDS-PAGE and WBs using Abs against GFP and α-tubulin as loading control. HRP-conjugated secondary Abs were used for enhanced chemiluminescence (ECL) detection in a Fusion FX6 Edge system (Vilber). For analysis of expression of Gα subunits, HEK293T (1 × 10^5^ cells/well) or HEK293T Ric8A-KO (3 × 10^5^ cells/well) cells were seeded on plates and transfected 48 hours later with 1 μg of total DNA using the following combinations: GFP-Ric8A/B (0.2 μg), Gα11/Gα13/Gαi1/Gαolf (0.4 μg), and wild-type Gαo or mutants (0.4 μg). After 24 hours, cells were lysed with ice-cold lysis buffer (20 mM Tris-HCl, pH 7.5, 100 mM NaCl, 5 mM MgCl_2_, 2 mM EDTA, 1% Triton X-100, 0.2% SDS, and 10% glycerol) supplemented with a protease inhibitor cocktail (Roche), and passed more than 10 times through a 25-G needle. Extracts were cleared by centrifugation at 15,000*g* and 4°C for 15 minutes, and analyzed as above using Abs against GFP, Gα subunits (Gα11, Gα13, Gαi1, Gαo, Gαolf), and α-tubulin. Quantification of all WBs was done using ImageJ v1.54f, and images were edited using EvolutionCapt v18.11 (Vilber) and CorelDRAW 2020.

### Co-IPs.

The recombinant GST-tagged nanobody against GFP (59) expressed in *E*. *coli* Rosettagami was purified with glutathione Sepharose 4B beads according to the manufacturer’s instructions. Protein purity was assessed by SDS-PAGE and Coomassie blue staining.

N2a (3 × 10^5^ cells /well) and HEK293T (2 × 10^5^ cells /well) cells were seeded on plates and cultured for 48 hours before cotransfection with 3 μg total DNA using the following combinations: Gαo-GFP and mRFP-Gβ1/Gγ3 (1 μg each), Gαo-GFP and His_6_-RGS19 (1.5 μg each), GFP-Gβ1 and mRFP-Gγ3 (1.5 μg each), GFP/GFP-Ric8A/B and nontagged Gα subunits (1.5 μg each), or GFP-Ric8A/B, nontagged Gαo, and Gα subunits (1 μg each). When indicated, DDD85646 was added to a 10 μM final concentration 7 hours after transfection (DMSO was used as control). After a 24-hour transfection, cells were resuspended with ice-cold GST-lysis buffer (20 mM Tris-HCl, pH 8.0, 1% Triton X-100, and 10% glycerol in PBS) supplemented with a protease inhibitor cocktail (Roche) and passed more than 10 times through a 25-G needle. Extracts were cleared by centrifugation at 15,000*g* for 15 minutes at 4°C, and supernatants were incubated with 2 μg of purified GST-tagged GFP nanobody for 30 minutes on ice. Then, 20 μL of glutathione Sepharose 4B beads were added, samples were rotated overnight at 4°C, beads were repeatedly washed with GST-lysis buffer, prepared for SDS-PAGE, and finally analyzed by WB using Abs against GFP, mRFP, His_6_-tag, Gαo, and the indicated Gα subunits, followed by incubation with HRP-conjugated secondary Abs for ECL detection, as mentioned above. If not quantified, co-IPs were done in duplicate with very similar outcomes.

### BRET assays.

The plasmid Go1-CASE encoding NLuc-tagged Gαo, Gβ3, and Venus-tagged Gγ9 was supplied by Gunnar Schulte (Karolinska Institutet, Stockholm, Sweden) (27). For the Gβ3γ9 displacement assay, HEK293T cells were cotransfected with the Go1-CASE plasmid and nontagged wild-type Gαo or mutants at a 1:1 ratio. For the M2R-based BRET, HEK293T cells were cotransfected with the M2R-NLuc plasmid, GFP-tagged Gαo, HA-tagged-Gγ3, and wild-type mRFP-Gβ1 or N88A/K89A mutant at a 2:2:1:1 ratio. Twelve hours after transfection, cells were resuspended in complete DMEM and seeded in transparent-bottom black 384-well plates (6,000 cells/well). After 24 hours, the medium was replaced with 10 μL of PBS, and furimazine was injected in an equal volume of PBS to a 10 μM final concentration immediately before measurement. GFP and NLuc signals were read at intervals of approximately 1.6 seconds using the built-in NanoBRET filter system for approximately 30 seconds, and ratios averaged. For M2R-NLuc-based BRET, acetylcholine (Sigma-Aldrich) was additionally injected afterwards in 10 μL volume to a final concentration of 10 μM, and signal changes were further recorded for approximately 50 seconds.

### Multiple sequence alignment.

The multiple sequence alignment for Ric8 proteins was done using the Clustal Omega tool of EMBL-EBI (60) and edited using Jalview 2.11.2.6 software (61). The following sequences were used: Ric8A *Mus musculus* (NP_444424.1), Ric8B *Mus musculus* (NP_898995.1), and dRic8 *Drosophila melanogaster* (NP_001285048.1).

### Statistics.

Data were analyzed using GraphPad Prism (v9.5.1). Data represent mean ± SEM or SD, as indicated. The differences between 1 group and a normalized control were analyzed by 1-sample *t* test, between 2 groups were analyzed by 2-tailed Mann-Whitney test, and multiple comparisons were analyzed by 1-way ANOVA followed by Dunnett’s multiple-comparison test or 2-way ANOVA followed by Šídák’s multiple-comparison test. Correlation analysis was performed using a 2-tailed Spearman’s correlation test. A *P* value of less than 0.05 was considered to be statistically significant; all *P* values are stated in figures and legends.

### Study approval.

Not applicable for this study.

### Data availability.

The data that support the findings of this study are available in the Supporting Data Values file or from the corresponding author upon request.

## Author contributions

MS and GPS performed molecular cloning. A Koval performed biochemical experiments, BRET analysis, and CRISPR/Cas9 cell line generation. GPS, JV, and A Kazemzadeh performed cellular experiments. GPS and VLK designed and supervised the study and wrote the manuscript. All authors read and approved the final manuscript.

## Supplementary Material

Supplemental data

Supporting data values

## Version 1 06/14/2024

In-Press Preview

## Version 2 08/01/2024

Electronic publication
