# Supplementary material for: Neomorphic Gαo mutations gain interaction with Ric8 proteins in GNAO1 encephalopathies
Source: J Clin Invest. 2024 Jun 14;134(15):e172057. doi: 10.1172/JCI172057 (PMC11291268; doi:10.1172/JCI172057)
Supplement: Supplemental data [file jci-134-172057-s185.pdf]

Neomorphic Gαo mutations gain interaction with Ric8 proteins in *GNAO1* encephalopathies

Gonzalo P. Solis[1]*, Alexey Koval[1], Jana Valnohova[1], Arghavan Kazemzadeh[1], Mikhail Savitsky[1], Vladimir L. Katanaev[1,2]*

*Corresponding authors: Gonzalo P. Solis and Vladimir L. Katanaev. Department of Cell Physiology and Metabolism, CMU,

Faculty of Medicine, University of Geneva. Rue Michel-Servet 1. CH-1211 Genève 4

Switzerland. Tel: +41 22 379 53 53. Emails: gonzalo.solis@unige.ch and vladimir.katanaev@unige.ch

Supplemental Material

Table S1. Clinical manifestations of the *GNAO1* encephalopathy mutations analyzed.

Online Mendelian Inheritance in Man (OMIM) entries for *GNAO1* encephalopathy: Developmental and Epileptic Encephalopathy-17 (**DEE17**; #615473) and Neurodevelopmental Disorder with Involuntary Movements (**NEDIM**; #617493).

Amino acid change	Nucleotide change	# of patients described	Age of onset	Epilepsy	Movement disorder (excl. hypotonia)	Developmental delay	Brain alterations (MRI)	Ref.	OMIM category and score (days of onset, mean ± sem)
G40R	c.118G>C<br>c.118G>A<br>c.118G>C<br>c.118G>A<br>c.118G>C	5	2.5m<br>birth<br>2m<br>6d<br>4m	yes<br>yes<br>yes<br>yes<br>yes	yes<br>no<br>yes<br>no<br>no	yes<br>yes<br>yes<br>N/A<br>yes	yes<br>no<br>yes<br>yes<br>yes	(1)<br>(2)<br>(3)<br>(4)<br>(5)	DEE17<br>52 ± 22 (n=5): Early
G45E	c.134G>A	1	4d	yes	yes	yes	yes	(6)	DEE17<br>4 (n=1): Very early
S47G	c.139A>G	1	4m	yes	yes	yes	yes	(3)	DEE17<br>120 (n=1): Late
Q52R	c.155A>G	1	1.5w	yes	yes	yes	yes	(7)	DEE17<br>10 (n=1): Early
D174G	c.521A>G	1	29d	yes	no	yes	yes	(8)	DEE17<br>29 (n=1): Early
L199P	c.596T>C	1	3d	yes	yes	yes	yes	(9)	DEE17<br>3 (n=1): Very early
G203R	c.607G>A<br><br><br><br><br><br><br><br>c.607G>A	9	7m<br>1d<br>12d<br>7m<br>7d<br>1m<br>3m<br>9d<br>12d	yes<br>yes<br>yes<br>yes<br>yes<br>yes<br>yes<br>yes<br>yes	yes<br>yes<br>yes<br>yes<br>yes<br>yes<br>yes<br>yes<br>yes	yes<br>yes<br>yes<br>yes<br>yes<br>yes<br>yes<br>yes<br>yes	yes<br>yes<br>yes<br>yes<br>yes<br>yes<br>yes<br>yes<br>no	(8)<br>(10)<br>(10)<br>(11)<br>(12)<br>(13)<br>(14)<br>(14)<br>(5)	DEE17<br>65 ± 29 (n=9): Early
R209C	c.625C>T<br>c.626G>A<br>c.625C>T<br>c.625C>T<br>c.625C>T<br>c.625C>T<br>c.625C>T<br>c.625C>T<br>c.625C>T<br>c.625C>T<br>c.625C>T<br>c.625C>T<br>c.625C>T<br>c.625C>T	13	6m<br>2y<br>infancy<br>6m<br>3-4m<br>2y<br>N/A<br>infancy<br>infancy<br>7m<br>1.5y<br>6m<br>birth<br>7m	no<br>no<br>no<br>yes<br>no<br>yes<br>yes<br>yes<br>yes<br>yes<br>no<br>no<br>yes<br>yes	yes<br>yes<br>yes<br>yes<br>yes<br>yes<br>yes<br>yes<br>yes<br>yes<br>yes<br>no<br>yes<br>yes	yes<br>yes<br>yes<br>yes<br>yes<br>yes<br>yes<br>yes<br>yes<br>yes<br>no<br>yes<br>yes<br>yes	yes<br>no<br>no<br>no<br>no<br>N/A<br>yes<br>yes<br>yes<br>yes<br>yes<br>yes<br>yes<br>no	(15)<br>(16)<br>(17)<br>(18)<br>(19)<br>(20)<br>(21)<br>(22)<br>(22)<br>(12)<br>(10)<br>(1)<br>(3)<br>(3)	NEDIM<br>305 ± 81 (n=10): Late
C215Y	c.644G>A	3	12y<br>5y<br>3y	no<br>no<br>no	yes<br>yes<br>yes	no<br>no<br>no	no<br>no<br>no	(23)<br>(24)<br>(24)	NEDIM<br>2400 ± 982 (n=3):<br>Very late

A227V	c.680C>T	2	2m	yes	yes	yes	yes	(12)	DEE17
			birth	yes	yes	yes	no	(25)	31 ± 30 (n=2): Early
Y231C	c.692A>G	3	5d	yes	no	yes	yes	(1)	DEE17
			3d	yes	yes	yes	no	(5)	23 ± 19 (n=3): Early
	c.692A>G		1m	yes	yes	yes	yes	(26)	
Q233P	c.698A>C	1	2y	no	yes	no	no	(27)	NEDIM
									720 (n=1): Very late
E237K	c.709G>A	8	6m	no	yes	yes	yes	(10)	NEDIM
	c.709G>A		3m	no	yes	yes	yes	(18)	666 ± 316 (n=5): Late
	c.709G>A		infancy	no	yes	yes	yes	(22)	
	c.709G>A		infancy	no	yes	yes	no	(22)	
	c.709G>A		6m	no	yes	yes	N/A	(28)	
	c.709G>A		4y	no	yes	yes	no	(29)	
	c.709G>A		4y	no	yes	yes	N/A	(29)	
	c.709G>A		N/A	no	yes	yes	N/A	(30)	
E246K	c.736G>A	13	4m	no	yes	yes	no	(12)	NEDIM
			11m	no	yes	yes	no	(14)	278 ± 109 (n=12): Late
			5m	yes	yes	yes	yes	(14)	
	c.736G>A		3m	no	yes	yes	yes	(18)	
			3m	no	yes	yes	yes	(25)	
	c.736G>A		3m	no	yes	yes	no	(31)	
	c.736G>A		3m	no	yes	yes	yes	(31)	
	c.736G>A		6m	no	yes	yes	yes	(31)	
	c.736G>A		5m	no	yes	yes	yes	(31)	
			9m	no	yes	yes	yes	(32)	
	c.736G>A		11m	no	yes	yes	no	(33)	
	c.736G>A		4y	no	yes	yes	yes	(33)	
	c.736G>A		childhood	yes	N/A	N/A	N/A	(34)	
N270H	c.808A>C	1	3m	yes	yes	yes	yes	(35)	DEE17
									90 (n=1): Early
F275S	c.824T>C	1	3d	yes	yes	no	yes	(35)	DEE17
									3 (n=1): Very early
I279N	c.836T>A	3	4d	yes	no	yes	yes	(8)	DEE17
	c.836T>A		1h	yes	yes	yes	yes	(1)	2 ± 1 (n=3): Very early
	c.836T>A		26min	yes	yes	yes	yes	(36)	

Table S2. Oligonucleotide primers used in this study.

Name	Sequence	Usage
Gαo-40-For	5’-TGCTCAGGGCTGGAGAATCAGG-3’	Generation of Gαo variants
Gao-40-Rev	5’-CCTGATTCTCCAGCCCTGAGCA-3’	
Gαo-45-For	5’-CTGGAGAATCAGAAAAAAGCACCATT-3’	
Gao-45-Rev	5’-AATGGTGCTTTTTTCTGATTCTCCAG-3’	
Gαo-47-For	5’-CTGGAGAATCAGGAAAAGGCACCATT-3’	
Gao-47-Rev	5’-AATGGTGCCTTTTCCTGATTCTCCAG-3’	
Gαo-174-For	5’-AGCAGGGCATCCTCCGAACCAG-3’	
Gao-174-Rev	5’-CTGGTTCGGAGGATGCCCTGCT-3’	
Gαo-199-For	5’-AGAACCTCCACTTCAGGCCGTTTG-3’	
Gao-199-Rev	5’-CAAACGGCCTGAAGTGGAGGTTCT-3’	
Gαo-227-For	5’-TGTGTCGTGCTCAGCGGCTATGACCAGGTGCT-3’	
Gao-227-Rev	5’-AGCACCTGGTCATAGCCGCTGAGCACGACACA-3’	
Gαo-231-For	5’-TGTGTCGCGCTCAGCGGCTGTGACCAGGTGCT-3’	
Gao-231-Rev	5’-AGCACCTGGTCACAGCCGCTGAGCGCGACACA-3’	
Gαo-233-For	5’-TGTGTCGCGCTCAGCGGCTATGACCCGGTGCT-3’	
Gao-233-Rev	5’-AGCACCGGGTCATAGCCGCTGAGCGCGACACA-3’	
Gαo-270-For	5’-TTCCTCCACAAGAAAGATCTCTTTGGCGAGAA-3’	
Gao-270-Rev	5’-TTCTCGCCAAAGAGATCTTTCTTGTGGAGGAA-3’	
Gαo-275-For	5’-TTCCTCAACAAGAAAGATCTCTCTGGCGAGAA-3’	
Gao-275-Rev	5’-TTCTCGCCAGAGAGATCTTTCTTGTTGAGGAA-3’	
Gαo-279-For	5’-TGGCGAGAAGAACAAGAAGTCACCT-3’	
Gao-279-Rev	5’-AGGTGACTTCTTGTTCTTCTCGCCA-3’	
dGαo-203-For	5’-TTACGTTCCGAGCGCTGACCGCGCACGTCAAACAATTTAA-3’	Generation of *Drosophila* Gαo G203R
dGao-203-Rev	5’-TTAAATTGTTTGACGTGCGCGGTCAGCGCTCGGAACGTAA-3’	
Gb1-N88A/K89A-For	5’-CTTATCATCTGGGACAGCTACACCACCgcCgcGGTCCACGCCATCCCTCTGCGCTCCTC-3’	Generation of Gβ1 mutant
Gb1-N88A/K89A-Rev	5’-GAGGAGCGCAGAGGGATGGCGTGGACCgcGgcGGTGGTGTAGCTGTCCCAGATGATAAG-3’	
CMV-For	5’-CGCAAATGGGCGGTAGGCGTG-3’	Generation of M2R-NLuc
M2R-AgeI-rev	5’-CTCTAGACACCGGTgcCCTTGTAGCGCCTA-3’	
Ric8A-SalI-For	5’-GCGTCGtCTTCgtcgacCCGGTGCCAGGGGCCATG-3’	Generation of GFP-Ric8A wild-type and mutants
Ric8A-PspOMI-Rev	5’-GGGAGCAgGGCCcCTGGCATCTTCAGTCAGGATCT-3’	
Ric8A-R75E-For	5’-CTATCCGAATCCTATCCgaAGACCGCAGCTGCCTGG-3’	
Ric8A-R75E-Rev	5’-CCAGGCAGCTGCGGTCTtcGGATAGGATTCGGATAG-3’	
Ric8A-K225A-For	5’-GTGATATTAAAGAGCACTgcGAGGATCTCCATGGCC-3’	
Ric8A-K225A-Rev	5’-GGCCATGGAGATCCTCgcAGTGCTCTTTAATATCAC-3’	
Ric8B-XhoI-For	5’-agcctgagctcgtttCTCgaGcggccgccaccatggatga-3’	Generation of GFP-Ric8B
Ric8B-EcoRI-Rev	5’-cctgtgtggcgaattctcagtctgtgtccgagctg-3’	
dRic8-BsrGI-For	5’-ATACAAGTTTGTACAAACAAGCAGGCTCGAGGGCCGCCGCCTTCACCATG-3’	Generation of *Drosophila* GFP-Ric8
dRic8-PspOMI-Rev	5’-CTGGGTCGGCGGGCCCACCCTAGGTTTTCCGCTTC-3’	

References

1. Kelly M, Park M, Mihalek I, Rochtus A, Gramm M, Pérez-Palma E, et al. Spectrum of neurodevelopmental disease associated with the GNAO1 guanosine triphosphate-binding region. *Epilepsia.* 2019;60(3):406-18.
2. Law CY, Chang ST, Cho SY, Yau EK, Ng GS, Fong NC, et al. Clinical whole-exome sequencing reveals a novel missense pathogenic variant of GNAO1 in a patient with infantile-onset epilepsy. *Clin Chim Acta.* 2015;451(Pt B):292-6.
3. Danti FR, Galosi S, Romani M, Montomoli M, Carss KJ, Raymond FL, et al. GNAO1 encephalopathy: Broadening the phenotype and evaluating treatment and outcome. *Neurol Genet.* 2017;3(2):e143.
4. Bruun TUJ, DesRoches CL, Wilson D, Chau V, Nakagawa T, Yamasaki M, et al. Prospective cohort study for identification of underlying genetic causes in neonatal encephalopathy using whole-exome sequencing. *Genet Med.* 2018;20(5):486-94.
5. Yang X, Niu X, Yang Y, Cheng M, Zhang J, Chen J, et al. Phenotypes of GNAO1 Variants in a Chinese Cohort. *Frontiers in Neurology.* 2021;12(637).
6. Gawlinski P, Posmyk R, Gambin T, Sielicka D, Chorazy M, Nowakowska B, et al. PEHO Syndrome May Represent Phenotypic Expansion at the Severe End of the Early-Onset Encephalopathies. *Pediatric Neurology.* 2016;60:83-7.
7. Solis GP, Kozhanova TV, Koval A, Zhilina SS, Mescheryakova TI, Abramov AA, et al. Pediatric Encephalopathy: Clinical, Biochemical and Cellular Insights into the Role of Gln52 of GNAO1 and GNAI1 for the Dominant Disease. *Cells.* 2021;10(10):2749.
8. Nakamura K, Kodera H, Akita T, Shiina M, Kato M, Hoshino H, et al. De Novo mutations in GNAO1, encoding a Galphao subunit of heterotrimeric G proteins, cause epileptic encephalopathy. *Am J Hum Genet.* 2013;93(3):496-505.
9. Marcé-Grau A, Dalton J, López-Pisón J, García-Jiménez MC, Monge-Galindo L, Cuenca-León E, et al. GNAO1 encephalopathy: further delineation of a severe neurodevelopmental syndrome affecting females. *Orphanet Journal of Rare Diseases.* 2016;11(1):38.
10. Schirinzi T, Garone G, Travaglini L, Vasco G, Galosi S, Rios L, et al. Phenomenology and clinical course of movement disorder in GNAO1 variants: Results from an analytical review. *Parkinsonism Relat Disord.* 2019;61:19-25.
11. Lee J, Park JE, Lee C, Kim AR, Kim BJ, Park W-Y, et al. Genomic Analysis of Korean Patient With Microcephaly. *Front Genet.* 2021;11:543528-.

12. Saitsu H, Fukai R, Ben-Zeev B, Sakai Y, Mimaki M, Okamoto N, et al. Phenotypic spectrum of GNAO1 variants: epileptic encephalopathy to involuntary movements with severe developmental delay. *Eur J Hum Genet.* 2016;24(1):129-34.
13. Arya R, Spaeth C, Gilbert DL, Leach JL, and Holland KD. GNAO1-associated epileptic encephalopathy and movement disorders: c.607G>A variant represents a probable mutation hotspot with a distinct phenotype. *Epileptic Disord.* 2017;19(1):67-75.
14. Schorling DC, Dietel T, Evers C, Hinderhofer K, Korinthenberg R, Ezzo D, et al. Expanding Phenotype of De Novo Mutations in GNAO1: Four New Cases and Review of Literature. *Neuropediatrics.* 2017;48(5):371-7.
15. Malaquias MJ, Fineza I, Loureiro L, Cardoso L, Alonso I, and Magalhães M. GNAO1 mutation presenting as dyskinetic cerebral palsy. *Neurol Sci.* 2019;40(10):2213-6.
16. Akasaka M, Kamei A, Tanifuji S, Asami M, Ito J, Mizuma K, et al. GNAO1 mutation-related severe involuntary movements treated with gabapentin. *Brain Dev.* 2021;43(4):576-9.
17. Kwong AK, Tsang MH, Fung JL, Mak CC, Chan KL, Rodenburg RJT, et al. Exome sequencing in paediatric patients with movement disorders. *Orphanet J Rare Dis.* 2021;16(1):32.
18. Waak M, Mohammad SS, Coman D, Sinclair K, Copeland L, Silburn P, et al. GNAO1-related movement disorder with life-threatening exacerbations: movement phenomenology and response to DBS. *J Neurol Neurosurg Psychiatry.* 2018;89(2):221-2.
19. Dzinovic I, Škorvánek M, Necpál J, Boesch S, Švantnerová J, Wagner M, et al. Dystonia as a prominent presenting feature in developmental and epileptic encephalopathies: A case series. *Parkinsonism Relat Disord.* 2021;90:73-8.
20. Danhofer P, Zech M, Bálintová Z, Baláž M, Jech R, and Ošlejšková H. Brittle Biballism-Dystonia in a Pediatric Patient with GNAO1 Mutation Managed Using Pallidal Deep Brain Stimulation. *Mov Disord Clin Pract.* 2021;8(1):153-5.
21. Chopra M, Gable DL, Love-Nichols J, Tsao A, Rockowitz S, Sliz P, et al. Mendelian etiologies identified with whole exome sequencing in cerebral palsy. *Annals of Clinical and Translational Neurology.* 2022;9(2):193-205.
22. Koy A, Cirak S, Gonzalez V, Becker K, Roujeau T, Milesi C, et al. Deep brain stimulation is effective in pediatric patients with GNAO1 associated severe hyperkinesia. *J Neurol Sci.* 2018;391:31-9.
23. Carecchio M, Invernizzi F, Gonzàlez-Latapi P, Panteghini C, Zorzi G, Romito L, et al. Frequency and phenotypic spectrum of KMT2B dystonia in childhood: A single-center cohort study. *Movement Disorders.* 2019;34(10):1516-27.

24. Wirth T, Tranchant C, Drouot N, Keren B, Mignot C, Cif L, et al. Increased diagnostic yield in complex dystonia through exome sequencing. *Parkinsonism Relat Disord.* 2020;74:50-6.

25. Kim SY, Shim Y, Ko YJ, Park S, Jang SS, Lim BC, et al. Spectrum of movement disorders in GNAO1 encephalopathy: in-depth phenotyping and case-by-case analysis. *Orphanet J Rare Dis.* 2020;15(1):343.

26. Talvik I, Møller RS, Vaher M, Vaher U, Larsen LH, Dahl HA, et al. Clinical Phenotype of De Novo GNAO1 Mutation:Case Report and Review of Literature. *Child Neurol Open.* 2015;2(2):2329048X15583717.

27. Yilmaz S, Turhan T, Ceylaner S, Gökben S, Tekgul H, and Serdaroglu G. Excellent response to deep brain stimulation in a young girl with GNAO1-related progressive choreoathetosis. *Childs Nerv Syst.* 2016;32(9):1567-8.

28. Okumura A, Maruyama K, Shibata M, Kurahashi H, Ishii A, Numoto S, et al. A patient with a GNAO1 mutation with decreased spontaneous movements, hypotonia, and dystonic features. *Brain Dev.* 2018;40(10):926-30.

29. Al Masseri Z, and AlSayed M. Gonadal mosaicism in GNAO1 causing neurodevelopmental disorder with involuntary movements; two additional variants. *Molecular Genetics and Metabolism Reports.* 2022;31:100864.

30. Fung EL, Mo CY, Fung ST, Chan AY, Lau KY, Chan EK, et al. Deep brain stimulation in a young child with GNAO1 mutation - Feasible and helpful. *Surg Neurol Int.* 2022;13:285.

31. Ananth AL, Robichaux-Viehoever A, Kim YM, Hanson-Kahn A, Cox R, Enns GM, et al. Clinical Course of Six Children With GNAO1 Mutations Causing a Severe and Distinctive Movement Disorder. *Pediatr Neurol.* 2016;59:81-4.

32. Takezawa Y, Kikuchi A, Haginoya K, Niihori T, Numata-Uematsu Y, Inui T, et al. Genomic analysis identifies masqueraders of full-term cerebral palsy. *Ann Clin Transl Neurol.* 2018;5(5):538-51.

33. Benato A, Carecchio M, Burlina A, Paoloni F, Sartori S, Nosadini M, et al. Long-term effect of subthalamic and pallidal deep brain stimulation for status dystonicus in children with methylmalonic acidemia and GNAO1 mutation. *J Neural Transm (Vienna).* 2019;126(6):739-57.

34. Helbig KL, Farwell Hagman KD, Shinde DN, Mroske C, Powis Z, Li S, et al. Diagnostic exome sequencing provides a molecular diagnosis for a significant proportion of patients with epilepsy. *Genet Med.* 2016;18(9):898-905.

35. De novo mutations in synaptic transmission genes including DNM1 cause epileptic encephalopathies. *Am J Hum Genet.* 2014;95(4):360-70.

36. De Novo Mutations in SLC1A2 and CACNA1A Are Important Causes of Epileptic Encephalopathies. *Am J Hum Genet.* 2016;99(2):287-98.

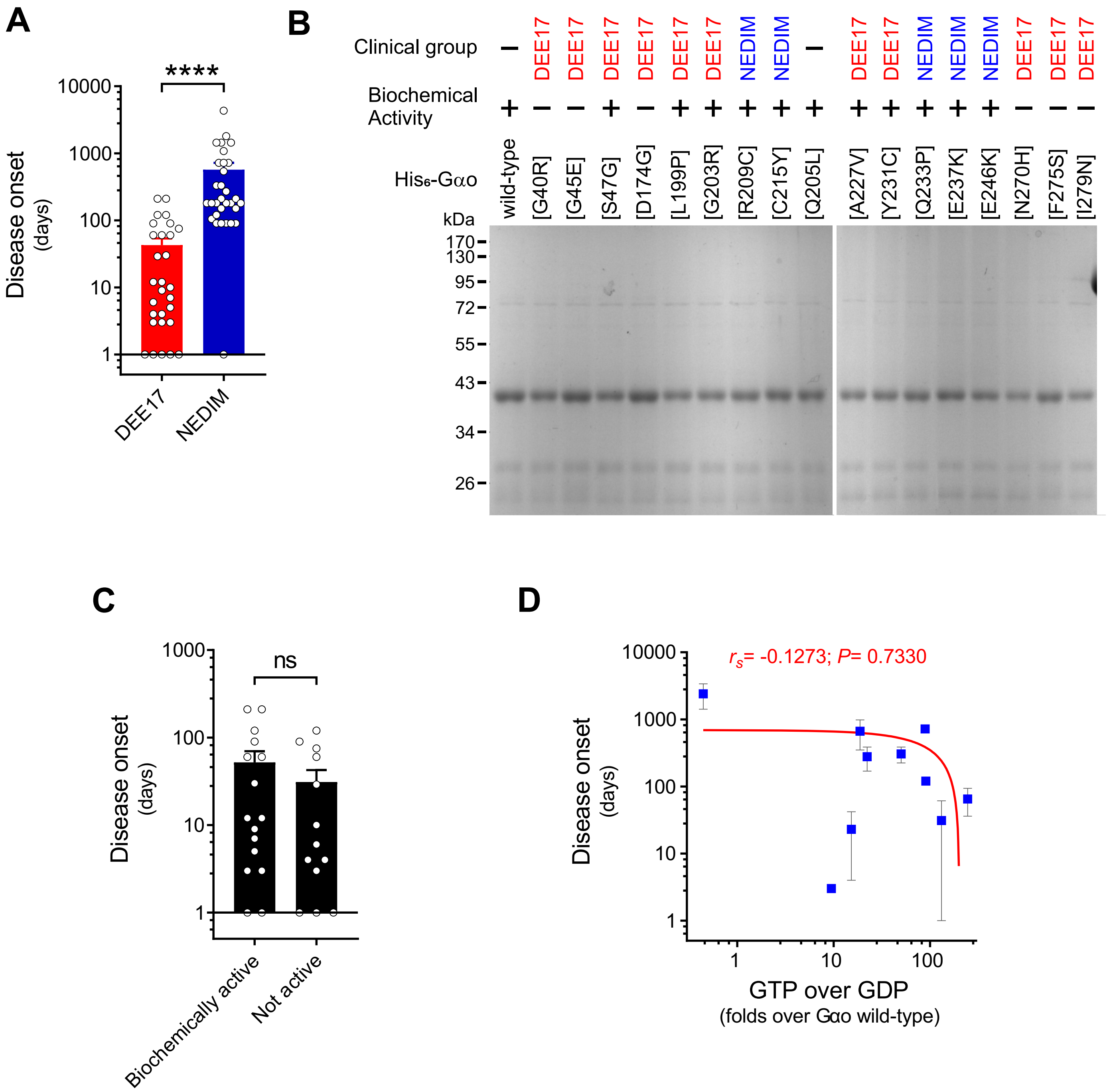

Supplemental Figure 1. Biochemical purification of Gαo encephalopathy mutants. (A) The Disease onset data from patients **(Supplemental Table S1)** was pooled according to the two classifications of *GNAO1* encephalopathy: Developmental and Epileptic Encephalopathy-17 (DEE17; red bars) and Neurodevelopmental Disorder with Involuntary Movements (NEDIM; blue bars). Note that the most severe DEE17 group shows in average a much lower Disease onset than the NEDIM group (n=29-31). **(B)** Coomasie blue staining of SDS-PAGE shows the purity of the recombinant His_6-tagged Gαo wild-type, encephalopathy mutants, and the control Q205L. The clinical manifestation associated to each Gαo mutant, and if they were purified active (+) or not active (-) is indicated. **(C)** The Disease onset data was grouped according to the biochemical activity of the recombinant Gαo mutants associated to the DEE17 disorder (n=13-16). **(D)** A scatterplot shows a non-significant negative correlation between Disease onset and the calculated GTP/GDP-loading ratio of Gαo variants. Note the log scale in the y axis. Data represent mean ± SEM. Data in **(A)** and **(C)** were analyzed by two-tailed Mann Whitney test, and in **(D)** by two-tailed Spearman correlation test (rank correlation coefficient (r_s) and P value are indicated). ns is not significant and $^{****}P < 0.0001$.

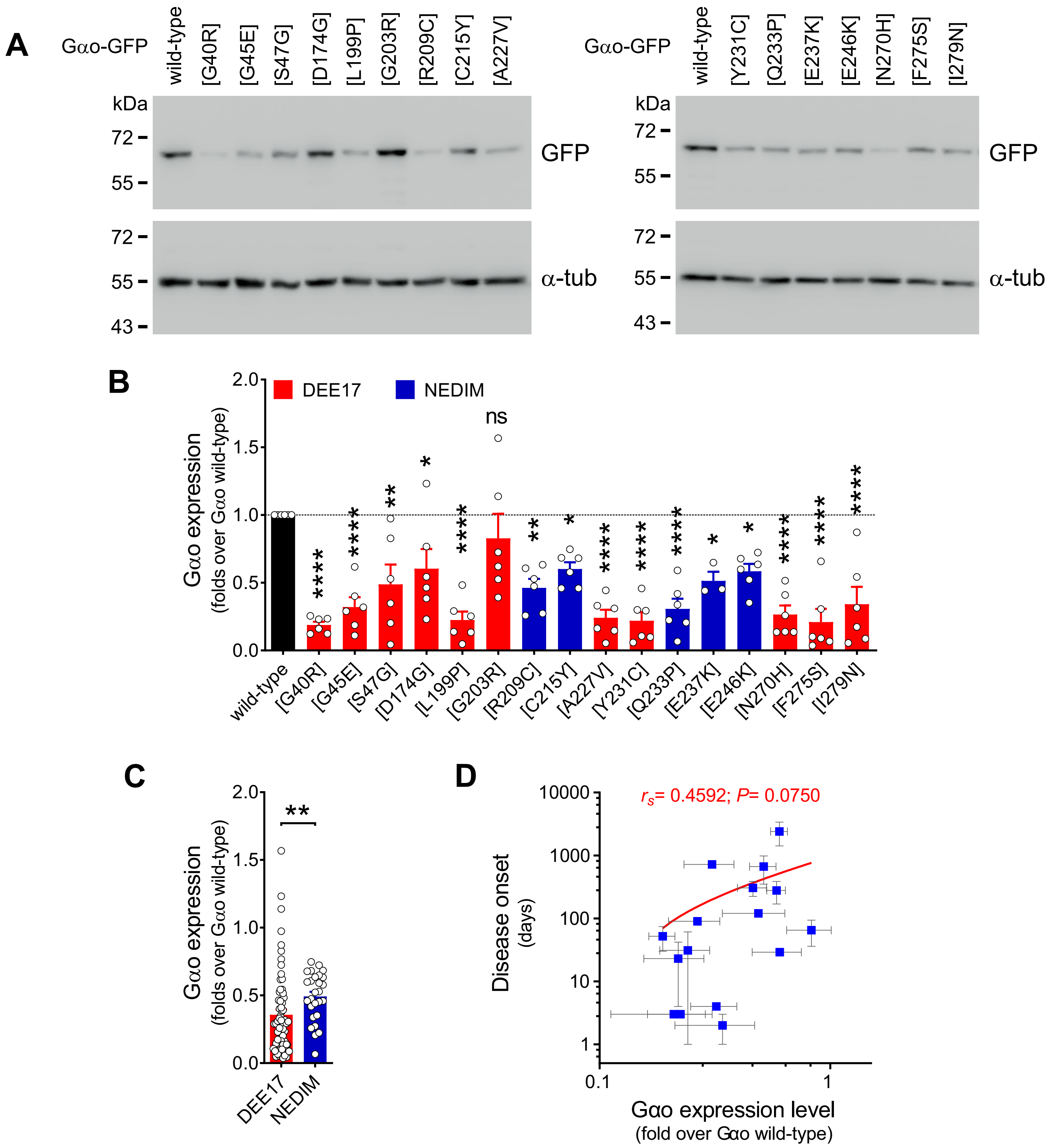

Supplemental Figure 2. Expression of Gαo encephalopathy mutants in N2a cells. **(A)** N2a cells were transfected with Gαo-GFP wild-type or encephalopathy mutants, and their expression levels were determined by Western blot using antibodies against GFP and against α-tubulin (α-tub) as loading control. **(B)** Quantification of the expression levels of Gαo variants (n=6). Data are color-coded according to the involvement of the mutants in Developmental and Epileptic Encephalopathy-17 (DEE17; red bars) or Neurodevelopmental Disorder with Involuntary Movements (NEDIM; blue bars). **(C)** The expression level of Gαo mutants pooled according to the DEE17 and NEDIM classification (n=27-66). **(D)** A scatterplot shows no significant correlation between Disease onset and the expression of Gαo variants. Note the log scale in the *y* axis. Data represent mean ± SEM. Data in **(B)** were analyzed by one-way ANOVA followed by Dunnett's multiple comparisons test, **(C)** by two-tailed Mann Whitney test, and **(D)** by two-tailed Spearman correlation test (rank correlation coefficient (r_s) and P value are indicated). ns is not significant, *$P < 0.05$, **$P < 0.01$, ***$P < 0.001$ and ****$P < 0.0001$.

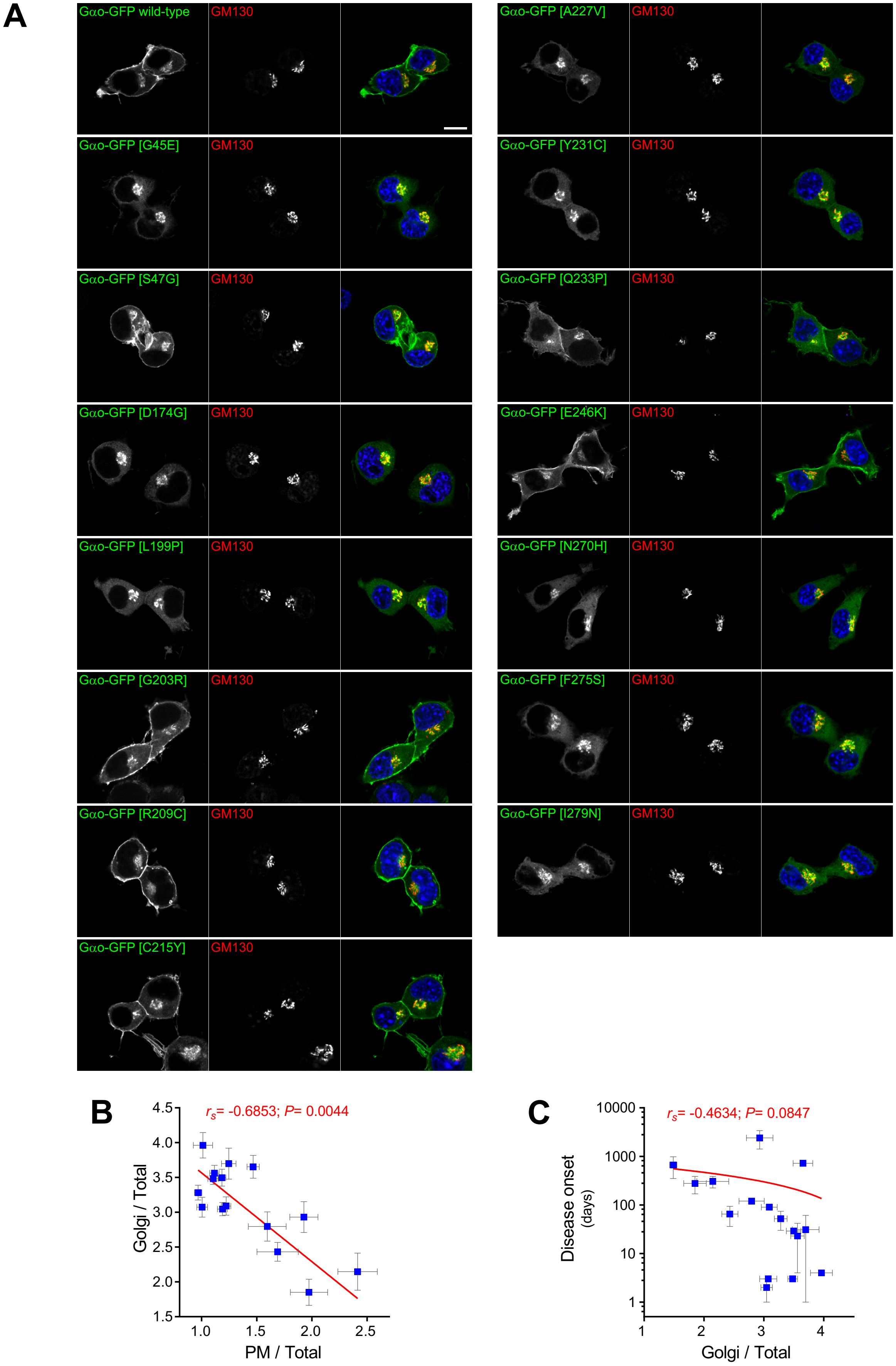

Supplemental Figure 3. Subcellular localization of Gαo mutants in N2a cells. (A) N2a cells expressing Gαo-GFP wild-type or the indicated mutants were immunostained against GM130 to visualize the Golgi apparatus. Scale bar, 10 μm. **(B)** A scatterplot shows a significant negative correlation between the relative localization of Gαo mutants at the plasma membrane (PM) and Golgi apparatus. **(C)** No significant correlation was calculated between Disease onset and the Golgi localization of Gαo variants. Note the log scale in the *y* axis. Data represent mean ± SEM. Data in **(B)** and **(C)** were analyzed by two-tailed Spearman correlation test; rank correlation coefficients (r_s) and *P* values are indicated.

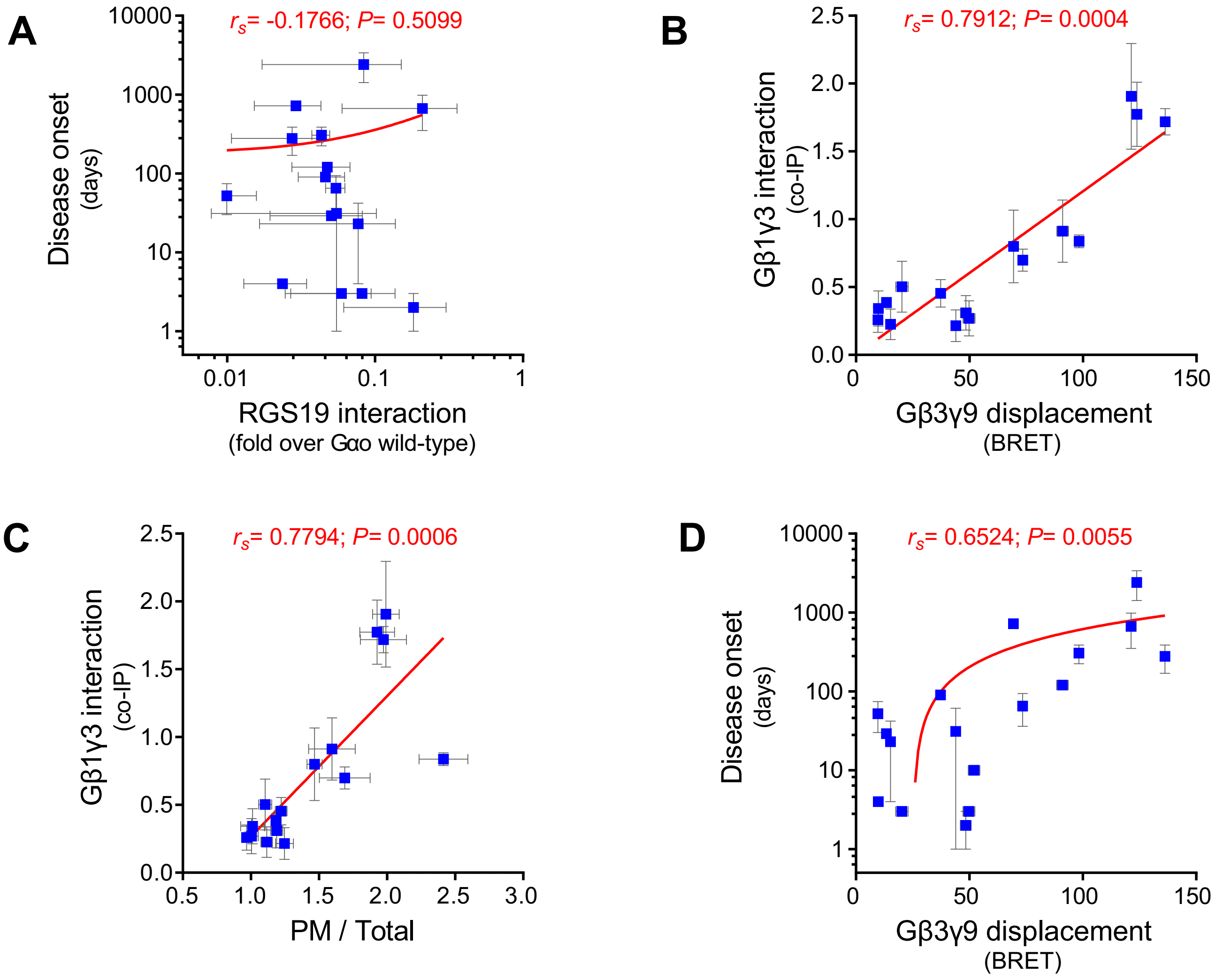

Supplemental Figure 4. Analysis of the cellular properties of Gαo encephalopathy mutants. **(A)** A scatterplot shows no significant correlation between Disease onset and RGS19 interaction of Gαo variants. Note the log scale in the *y* axis. **(B** and **C)** Strong positive correlations were calculated between Gβ1γ3 interaction and Gβ3γ9 displacement **(B)** and plasma membrane (PM) localization **(C)** of Gαo mutants. **(D)** A significant positive correlation is also seen between Disease onset and Gβ3γ9 displacement. Data represent mean ± SEM. All data were analyzed by two-tailed Spearman correlation test; rank correlation coefficients (r_s) and P values are indicated.

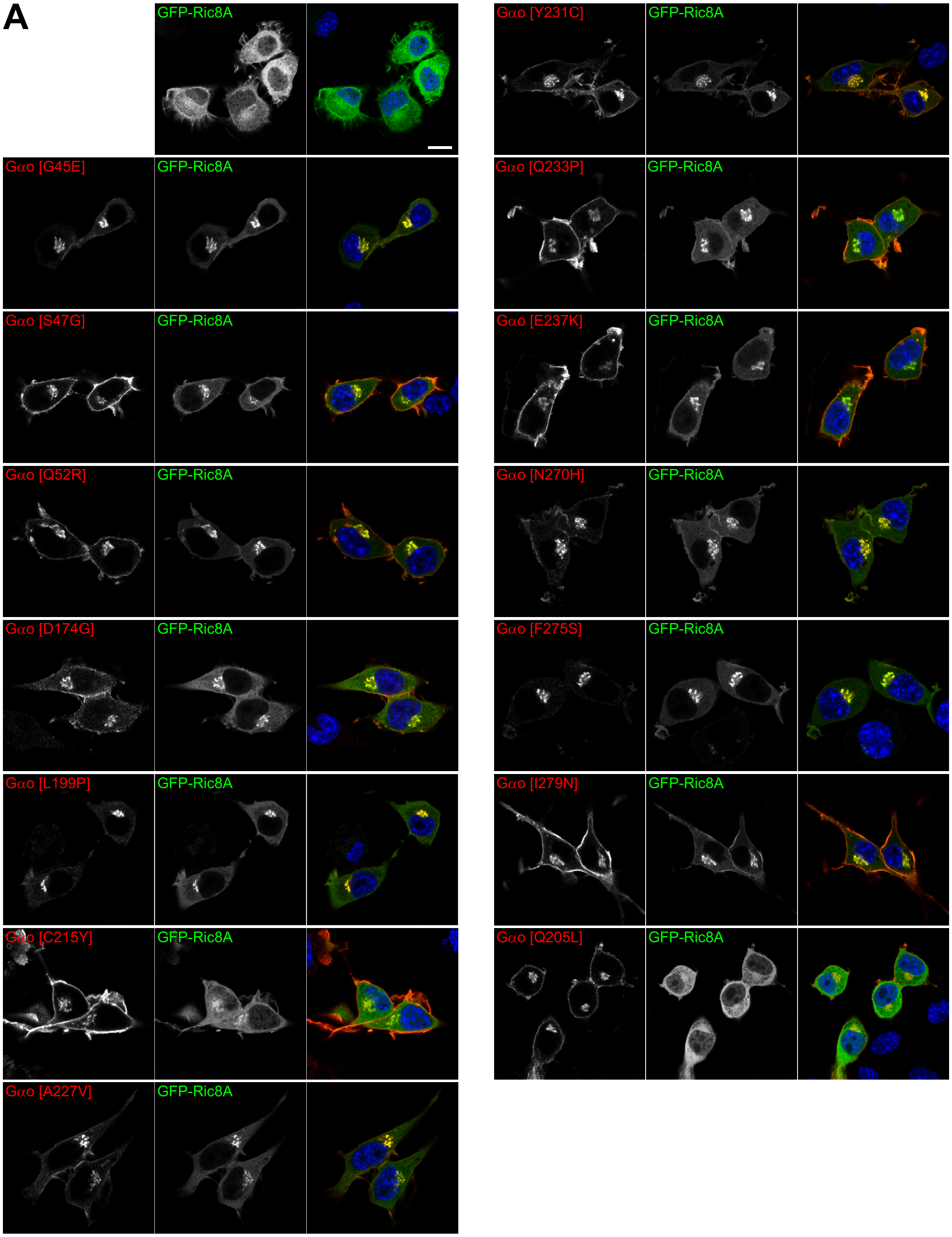

Supplemental Figure 5. Golgi delocalization of Ric8A by Gαo encephalopathy mutants. (A) Representative images of N2a cells expressing GFP-Ric8A alone, together with Gαo encephalopathy mutants, or the GTPase-deficient Q205L mutant as control. Note that the normal cytoplasmic localization of Ric8A is drastically shifted to the Golgi (and to a lesser extent to the plasma membrane) by the co-expression of Gαo encephalopathy variants, but not Q205L. Gαo was detected by immunostaining using an specific antibody. Scale bar, 10 μm.

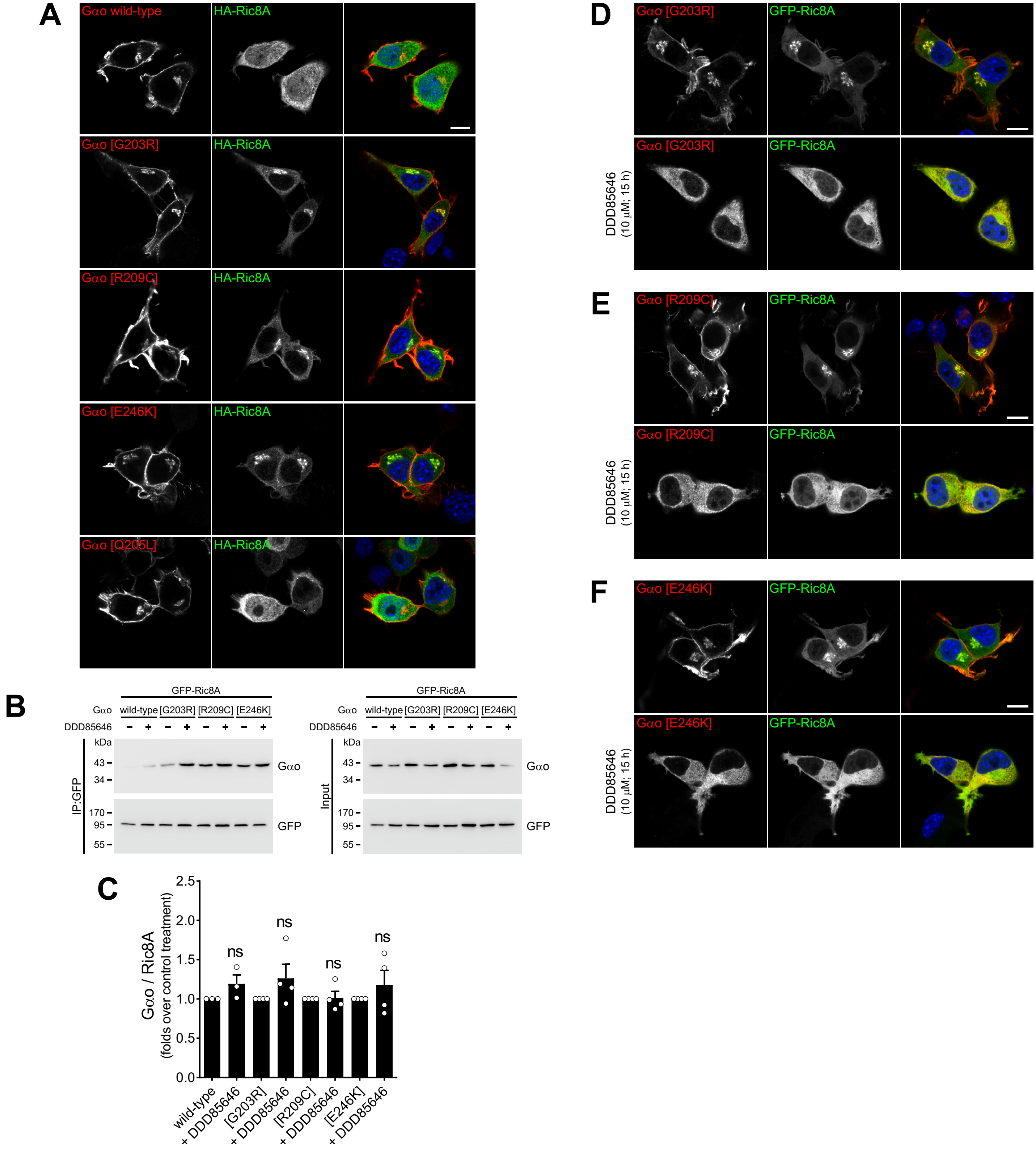

Supplemental Figure 6. Golgi delocalization of Ric8A by encephalopathy mutants depends on Gαo lipidations. (A) N2a cells co-expressing HA-tagged Ric8A (HA-Ric8A) together with Gαo wild-type, the mutants G203R, R209C, E246K, or the control Q205L were immunostained against Gαo and the HA-epitope, and stained with DAPI in blue for nuclei. Note the strong cytoplasm-to-Golgi delocalization of Ric8A only in the presence of encephalopathy mutants. **(B)** Immunoprecipitation (IP) of GFP-Ric8A was done from N2a cells preincubated for 15 h with 10 µM of the N-myristoylation blocker DDD85646 (+) or DMSO as control (-), and using a nanobody against GFP. The co-precipitation of Gαo variants was determined by immunodetection with antibodies against Gαo and GFP. **(C)** Quantification of Gαo co-IP reveals no significant effect of the N-myristoylation inhibitor (n=3-4). **(D–F)** Representative images of N2a cells showing that the Golgi delocalization of Ric8A and overall membrane association of Gαo variants were abolished by the DDD85646 treatment. Scale bars, 10 µm. Data represent mean ± SEM. The data in **(C)** were analyzed by one-way ANOVA followed by Dunnett's multiple comparisons test; ns is not significant.

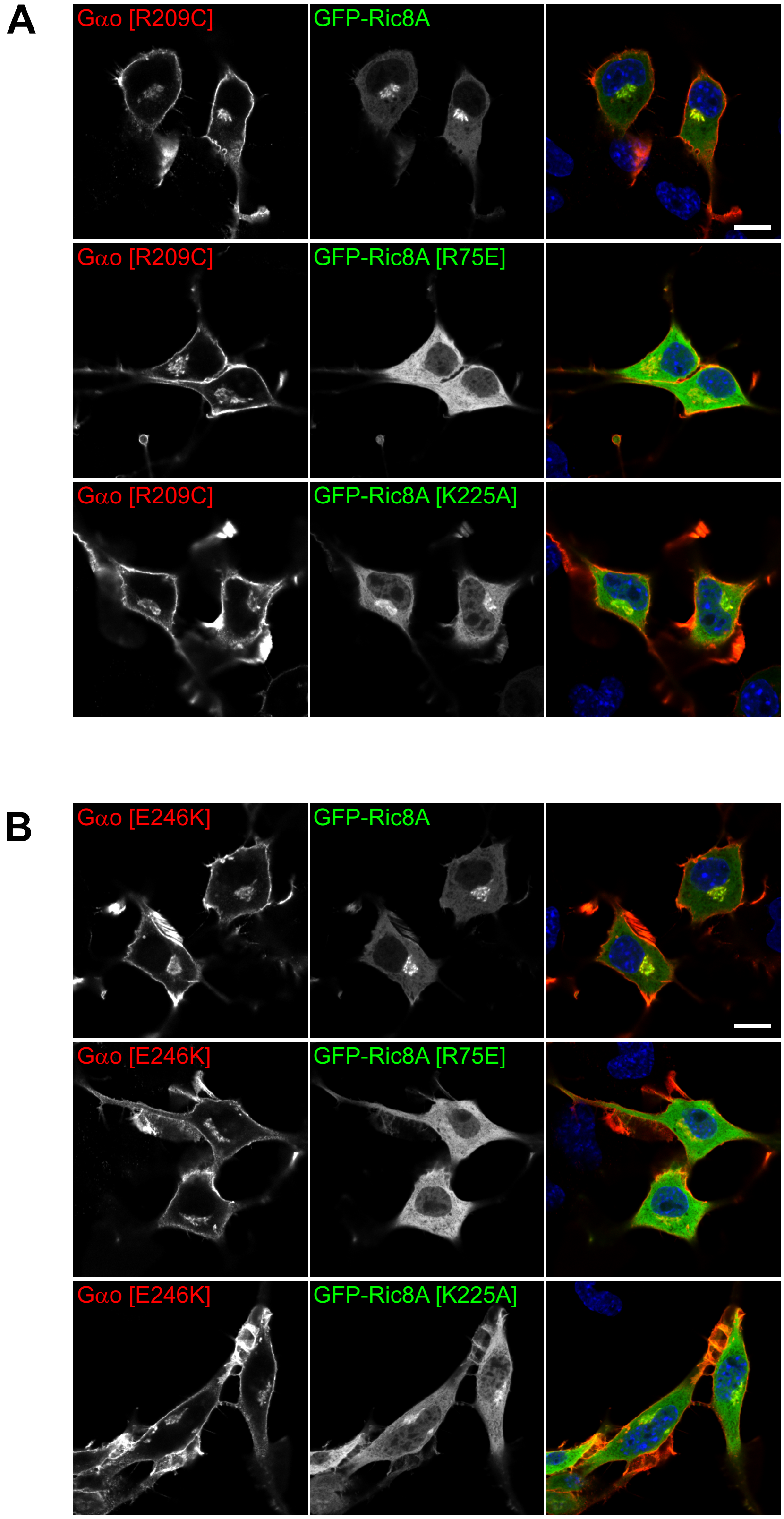

Supplemental Figure 7. The neomorphic Ric8A interaction of Gαo encephalopathy mutants depends on Ric8A chaperone activity. (**A** and **B**) N2a cells co-expressing the GFP-Ric8A and Gαo constructs indicated in the panels were immunostained against Gαo, and DAPI staining in blue indicates nuclei. Note that the strong Golgi-delocalization of Ric8A by the Gαo R209C and E246K mutants is clearly reduced or lost for the chaperone-deficient mutants K225A and R75E, respectively. Scale bars, 10 µm.

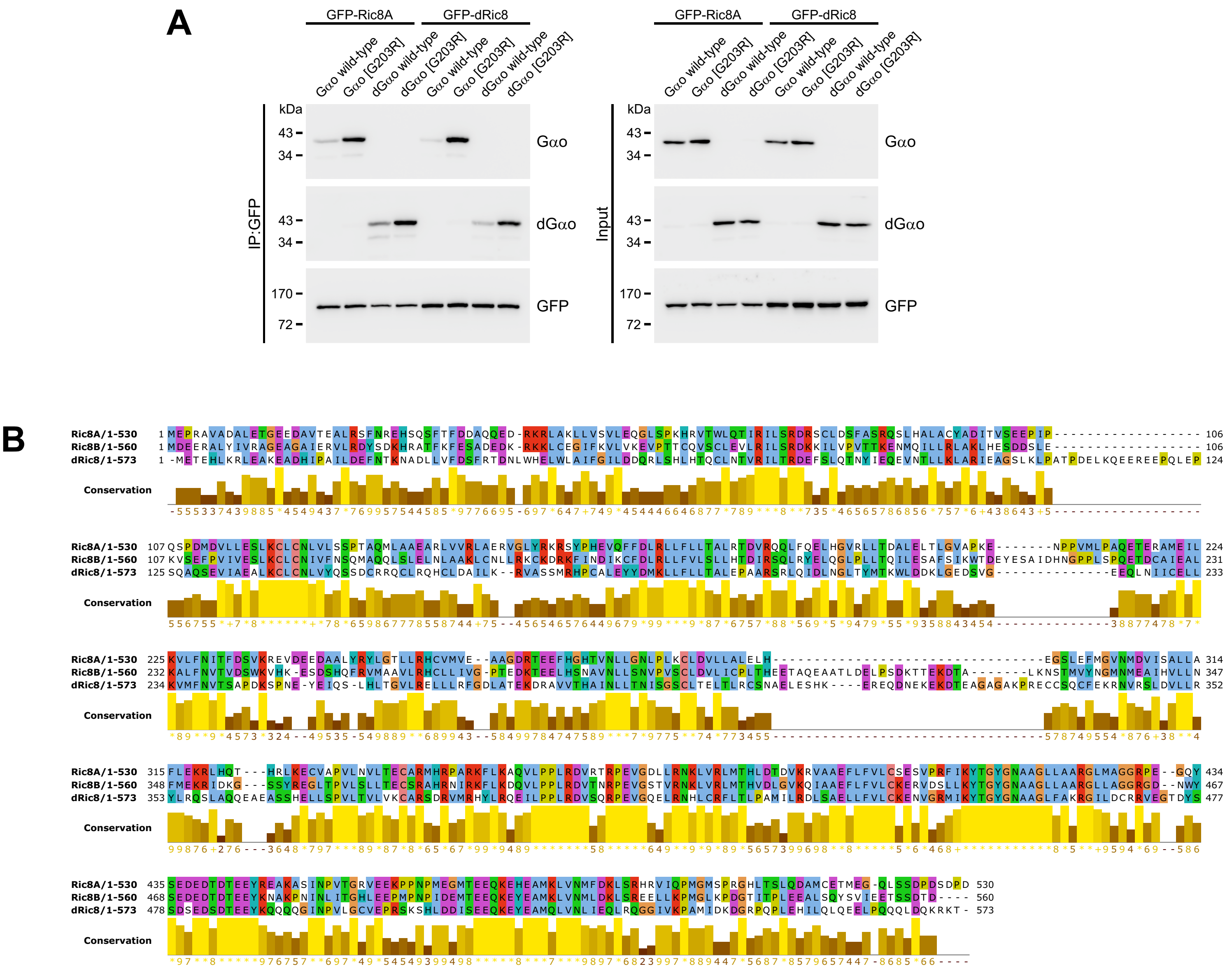

Supplemental Figure 8. The neomorphic Ric8-Gαo mutant interaction is conserved between fly and mammals. (A) N2a cells were co-transfected with GFP-Ric8A (mouse) or GFP-dRic8 (*Drosophila*), and the G203R mutant of Gαo (human) and dGαo (*Drosophila*). The immunoprecipitation (IP) of GFP constructs was done with a nanobody against GFP and analyzed by Western blot using antibodies against GFP, Gαo, and dGαo. **(B)** A multiple sequence alignment of Ric8 proteins including Ric8A *Mus musculus* (NP_444424.1), Ric8B *Mus musculus* (NP_898995.1), and dRic8 *Drosophila melanogaster* (NP_001285048.1).

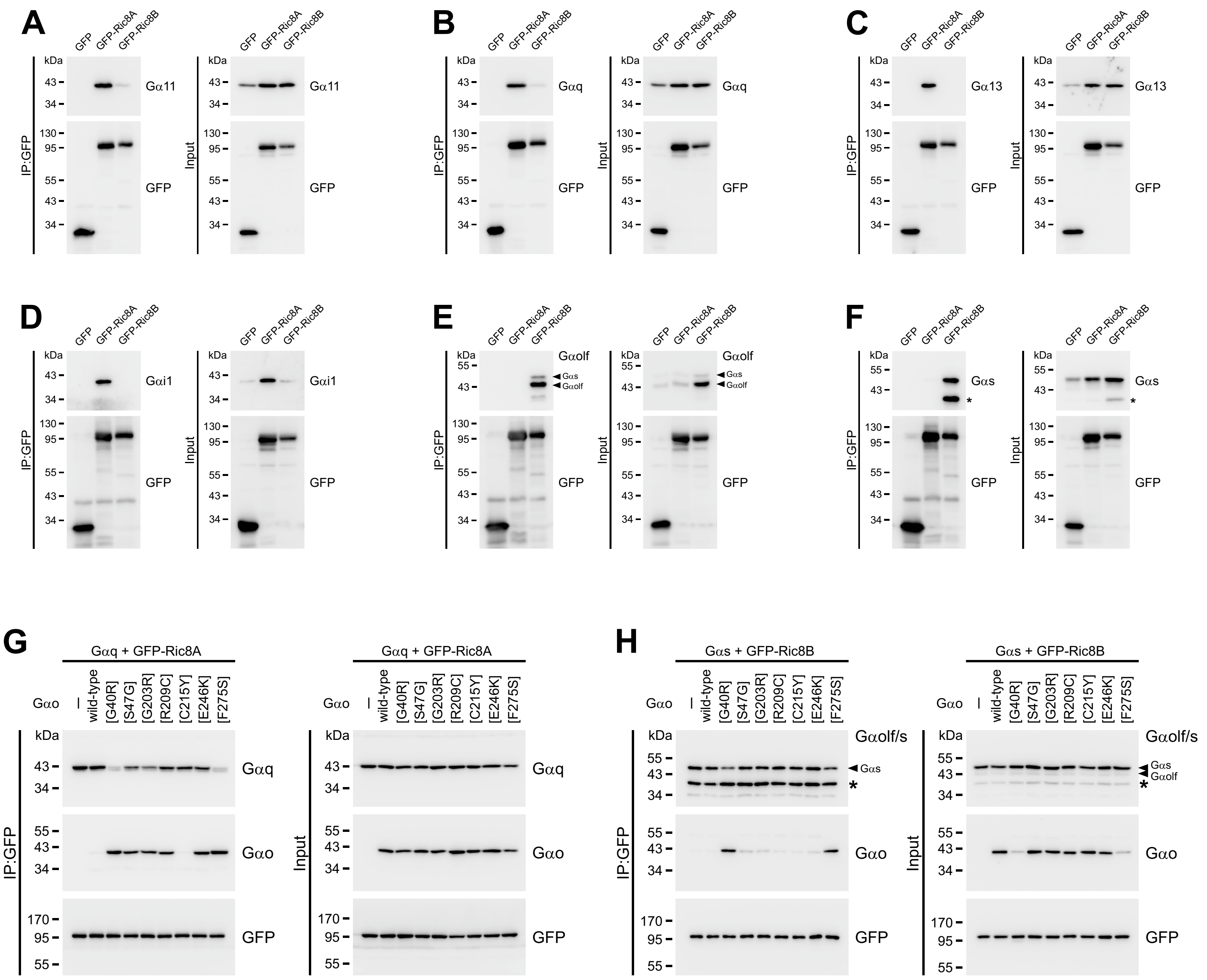

Supplemental Figure 9. Ric8A/B interaction with Gα subunits. **(A–F)** HEK293T cells were co-transfected with GFP, GFP-Ric8A or GFP-Ric8B and non-tagged Gα11 **(A)**, Gαq **(B)**, Gα13 **(C)**, Gαi1 **(D)**, Gαolf **(E)**, or Gαs **(F)**. The immunoprecipitation (IP) of GFP proteins was done using a nanobody against GFP and the interaction with the Gα subunits was determined by Western blot. Immunodetection was achieved using antibodies against GFP, Gαo, Gα11, Gαq, Gα13, Gαi1, and Gαolf/s. **(G** and **H)** HEK293T cells were co-transfected with GFP-Ric8A and Gαq **(G)** or GFP-Ric8B and Gαs **(H)**, and Gαo wild-type, mutants or empty plasmid (-). The IP of GFP-Ric8A/B was done and analyzed as above. Arrowheads point to Gαolf and Gαs, and (*****) indicates a prominent degradation product of Gαs.

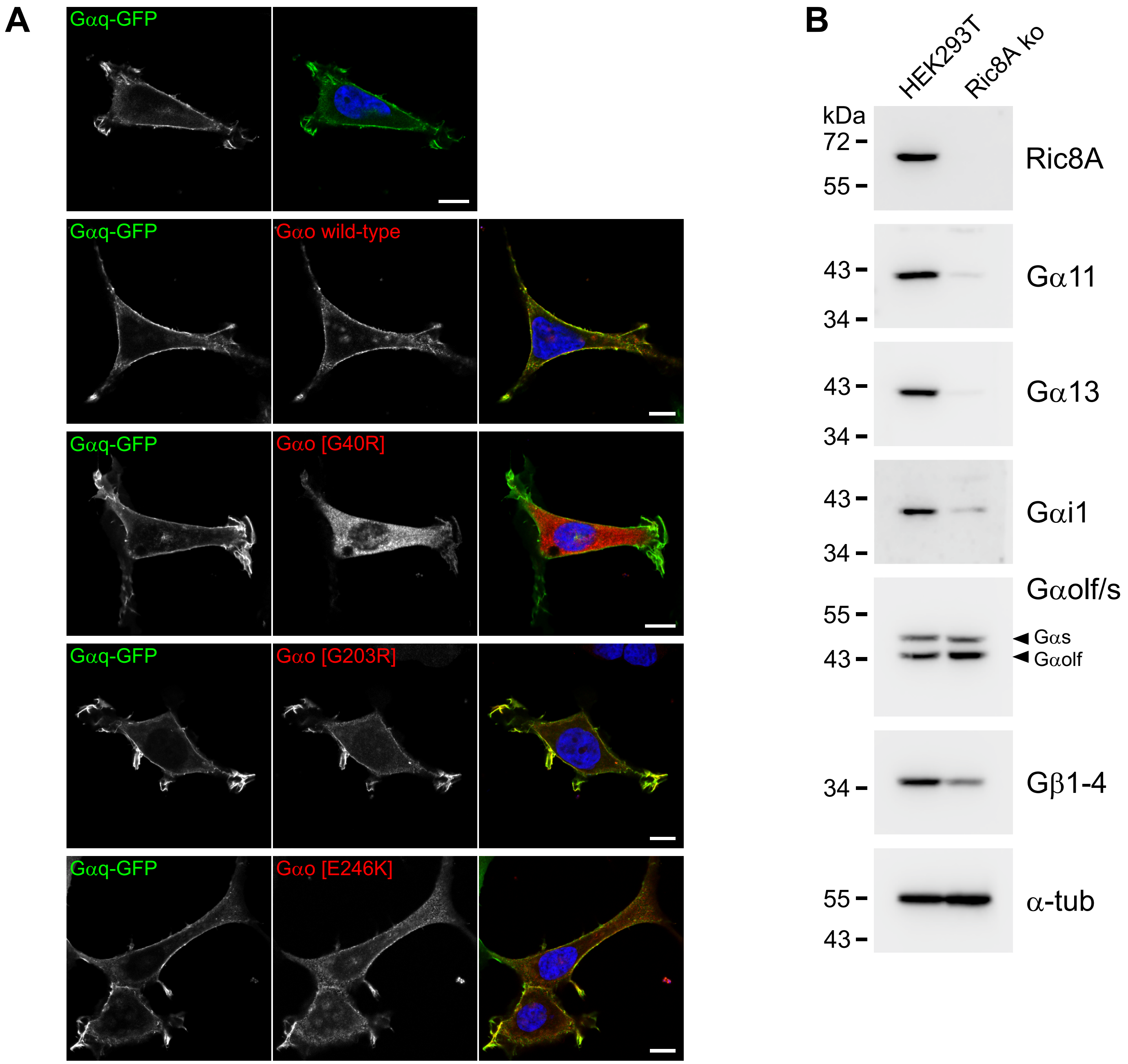

Supplemental Figure 10. PM localization of Gαq is not blocked by Gαo encephalopathy mutants. **(A)** HEK293T cells expressing Gαq-GFP alone or together with Gαo wild-type, G40R, G203R, or E246K were immunostained against Gαo and stained with DAPI to visualized nuclei in blue. Note that Gαq is efficiently targeted to the PM in the presence of Gαo variants. Scale bars, 10 µm. **(B)** HEK293T Ric8A knockout (ko) cells were analyzed by Western blot alongside the HEK293T parental line. Immunodetection was done using antibodies against Ric8A, Gα11, Gα13, Gαi1, Gαolf/s, Gβ1-4, and α-tubulin (α-tub) as loading control.
